# Assessment of machine learning models trained by molecular dynamics simulations results for inferring ethanol adsorption on an aluminium surface

**DOI:** 10.1038/s41598-024-71007-z

**Published:** 2024-09-03

**Authors:** Fatemeh Shahbazi, Mohammad Nasr Esfahani, Amir Keshmiri, Masoud Jabbari

**Affiliations:** 1 Warwick Manufacturing Group (WMG), University of Warwick, Coventry, CV4 7AL UK; 2 School of Engineering, University of Manchester, Manchester, M13 9PL UK; 3 School of Physics, Engineering and Technology, University of York, York, YO10 5DD UK; 4 School of Mechanical Engineering, University of Leeds, Leeds, LS2 9JT UK

**Keywords:** Mechanical engineering, Computational science

## Abstract

Molecular dynamics (MD) simulations can reduce our need for experimental tests and provide detailed insight into the chemical reactions and binding kinetics. There are two challenges while dealing with MD simulations: one is the time and length scale limitations, and the latter is efficiently processing the massive amount of data resulting from the MD simulations and generating the proper reaction rates. In this work, we evaluated the use of regression machine learning (ML) methods to solve these two challenges by developing a framework for ethanol adsorption on an Aluminium (Al) slab. This framework comprises three main stages: first, an all-atom molecular dynamics model; second, ML regression models; and third, validation and testing. In stage one, the adsorption of ethanol molecules on the Al surface for various temperatures, velocities and concentrations is simulated using the large-scale atomic/molecular massively parallel simulator (LAMMPS) and ReaxFF. The outcome of stage one is utilised for training, testing, and validating the predictive models in stages two and three. We developed and evaluated 28 different ML models for predicting the number of adsorbed molecules over time, including linear regression, support vector machine (SVM), decision trees, ensemble, Gaussian process regression (GPR), neural network (NN) and Bayesian hyper-parameter optimisation models. Based on the results, the Bayesian-based GPR showed the highest accuracy and the lowest training time. The developed model can predict the number of adsorbed molecules for new cases within seconds, while MD simulations take a few weeks. This adsorption rate can then be used in macroscale simulations to tackle the time and length scale limitations. The proposed numerical framework has the potential to be generalised and, therefore, contribute to future low-cost binding reaction estimations, providing a valuable tool for industry and experimentalists.

## Introduction

Aluminium (Al) is widely used in energetic applications as the reactive metal for propulsion and energy conversion^1^ due to two main reasons. Firstly, in comparison to the other metallic materials, Al exhibits high exothermic (release) energy of 31.05 kJ g $^{-1}$ during oxidation reaction^2^. Secondly, its reaction products are not toxic to the atmosphere^3^. However, storing and protecting Al in an oxygen environment is challenging. Hence, different coating methods using organic and inorganic materials are used to ensure their safety. Organic coating helps to improve the enthalpy of combustion and provides an entire coating and preservation of the metal^4^. Ethanol is mainly used as a protective organic coating solution for long-term storage. Understanding the adsorption behaviour of ethanol on the surface of Al slab in different conditions is important for improving the coating process. Although there have been numerous experimental studies, few have delved into the specific interactions between Al and the coating material at the molecular level^5^. In this section, we first review the current development in numerical simulations of binding reactions with a focus on adsorption of ethanol on an Al slab. As significant computational costs are linked to the molecular dynamics (MD) simulations, researchers are using the large amount of data generated by the MD simulations to train machine learning (ML) models and improve the capabilities in predicting the adsorption process^6,7^. Hence, we review the current use of ML models to accelerate MD simulations in the second part and explore the features of Bayesian optimisation and GPR methods, as well as their potential for predicting complex behaviours.

### Numerical simulations of molecular adsorption of ethanol on an Al slab

Computational methods are helpful tools for complementing experimental techniques  ^8,9^. Due to the complexity of the binding reactions and particle behaviour in different environment conditions, numerical simulations can provide valuable insights. With this aim, researchers are developing various numerical solutions utilising molecular docking, virtual screening, hybrid approaches, quantum mechanics (QM) calculations and MD simulations  ^10^. Molecular docking predicts the most probable matching mode of a ligand  ^11^, and virtual screening  ^12^ analyses the receptor-based and ligand-based approaches with the docking of the library. While QM methods provide accurate information regarding the mechanical and chemical changes in the atomic scale, the time and length scales are significantly limited  ^13^, around 10 ps and 10 nm, respectively. In MD simulations, atoms are modelled as point particles, finite-size spheres, ellipsoids or triangles. MD can simulate the atomic scale (all-atom model) and coarse-grained up to meso/continuum scale and 10 ms. Usually, MD simulation utilises orthogonal or triclinic (skewed) simulation cells. MD simulations can reproduce the QM results and scale up to millions to billions of atoms. MD methods are not just limited to the interaction of the system of atoms; complicated methods can solve mechanics, material science and biophysics (proteins, brain cells)  ^14^.

In previous works, the adsorption of ethanol on Al slab^5^ and Al nanoparticles^3,15, 16^ has been successfully simulated using MD methods. In these works, they developed a reactive force-field (ReaxFF^17^) for Al, carbon and oxygen inter-atomic potential^18^. ReaxFF implements the distance-dependent bond-order function to represent the contribution of chemical bonding to the potential energy. This method estimates the bond orders ( $BO_{ij}$ ) between atoms from their inter-atomic distances (Eq. 1)  ^5^.

1 $$\begin{aligned} \begin{matrix} BO_{ij}=BO_{ij}^\varrho +BO_{ij}^\pi +BO_{ij}^{\pi \pi }= exp\Big [p_{bo1}\Big (\frac{r_{ij}}{r_o^\varrho }\Big )^{P_{bo2}}\Big ]+exp\Big [p_{bo3}\Big (\frac{r_{ij}}{r_o^\pi }\Big )^{P_{bo4}}\Big ]+exp\Big [p_{bo5}\Big (\frac{r_{ij}}{r_o^{\pi \pi }}\Big )^{P_{bo6}}\Big ] \end{matrix} \end{aligned}$$

where $BO_{ij}$ is the bond order between atoms *i* and *j*, $r_{ij}$ is their inter-atomic distance. The terms $\varrho$, $\pi$, and $\pi \pi$ are the bond characters, $r_o$ is the equilibrium bond length and $p_{bo}$ terms are the empirical parameters. For over coordination ($\Delta _i >0$) and under coordination ($\Delta _i <0$ estimations, Eq. (2) is used. It calculates the difference between the total bond order around the atom and the number of its bonding electrons.

2 $$\begin{aligned} \acute{\Delta } i= -Val_i+ \sum _{j=1}^{neighbours(i)} \acute{BO}_{ij} \end{aligned}$$

where $Val_i$ is the number of bonding electrons, and $\acute{\Delta }i$ is the over coordination^19^. Hong et al. validated the ReaxFF parameters for Al, Carbon, Oxygen, and hydrogen interactions with quantum mechanics (QM) calculations and experimental data^16^. Hence, the ReaxFF developed by S. S. Hong *et al.* were used as an input for the numerical simulations for this project without the need for extra validation or QM simulations.

### Bayesian regression ML methods for analysing the MD simulations data

Despite the successful simulation of the adsorption of ethanol on Al surfaces, MD has time and length scale limitations. ML can help improve molecular interaction simulation to tackle these limitations^20,21^. ML is a data-driven approach that offers a structured framework for mapping complex processes. Due to its self-learning ability and fast predictions, it can replace the classical models and give us more ability for prediction^21,22^. These prediction models are generated without the previous need for physics, although recently, there has been a focus on developing physics-based ML methods for generalisation and higher accuracy. In this work, we focus on non-physical ML methods.

Molecular dynamics simulations and ML are complementary in interpreting ambiguous experimental data and obtaining structural models. For instance, the FLAPS platform finds functional parameters in X-ray scattering protein simulations^23^. Deep learning and highly parallelisable graphics processing units (GPUs) computing have also benefited drug discovery^24^. The transmute framework applies to vaccine design and optimisation. The current vaccine, drug and biosensor design methods are not yet automated^25^. Another key challenges in such numerical simulations is their reliance on the MD parameters^26^. The solution for this challenge is Bayesian methods, which drive a proper weighting^27^. Bayesian approach is based on Bayes’ theorem and finds the probability of the parameters of the model ($\theta$) after the data observation  ^28^. Hence, the Bayesian model provides more insight into the data and a more intelligent prediction model. Bayesian is an optimal method for updating these beliefs about $\theta$ based on the new information  ^29^.

After reviewing prior research, it appears that there is a shortage of benchmarks for evaluating various ML methods trained using MD simulation data of Ethanol adsorption on Al slab and testing their ability to predict binding reactions, which is the primary focus of this study. We made sure to include potential machine learning models for predicting complex behaviours, such as Gaussian process regression (GPR), in this benchmark. ML methods usually apply two common approaches: restriction bias and preference bias. The first one restricts the class of functions based on the input, which provides poor predictions if the target function is not well-modelled by the chosen class. As a solution, the class function’s flexibility is usually increased, which leads to over-fitting^30^. In the preference bias approach, a prior probability is assigned to every possible function, whereas higher probabilities are dedicated to the functions that seem to be more likely. This approach also has the problem of giving an infinite set of possible functions, which would be impossible to solve in a finite time. The GPR is the solution. It is a generalisation of the Gaussian probability distribution^31^.

### Aim and objectives

This work aims to evaluate different ML models for inferring the number of adsorbed ethanol on an Al slab for various conditions, using the MD simulation results as the training data set. Utilising ML with MD simulation results will reduce the need for experimental inputs for the computational simulation of binding reactions and overcome the time and length scale limitation in nanoscale studies. We first introduce the three stages of the numerical framework and then analyse the MD simulation results and evaluate the ML predictions. The numerical framework consists of molecular dynamics simulations and the training, validation and testing of 28 different Bayesian regression ML models. In previous attempts, the molecular interaction of the solvent and surface is studied without considering the fluid flow behaviour of the solvent  ^5^, which is considered in the current MD simulation study.

## Methods

The numerical framework developed in this work consists of three main stages: molecular dynamics simulation of ethanol molecule on an Al slab, training regression ML models for inferring the number of adsorbed molecules, and validation and testing. This framework takes the surface and analyte molecule specifications as input. It simulates the binding reactions of the molecules passing on top of the surface and develops a prediction model for the binding reactions.

### Stage 1: molecular dynamics simulation of ethanol molecule on an Al slab

In stage one, a reactive atomic simulation has been carried out, predicting the binding properties of the target species and the Al slab^32,33^. The large-scale atomic/molecular massively parallel simulator (LAMMPS^34^)  ^35^ is used for this molecular simulation. The boundary vertical to the surface is set as fixed (*z*), and the two other directions (*x* and *y*) are defined as periodic. Periodic boundary conditions reduce finite-size effects and simulate bulk conditions. Using the OVITO program^36^, the simulation box and the nano-Al block are developed. Equation (3) is used to calculate the overall system energy for the MD simulation.

3 $$\begin{aligned} \begin{matrix} E_{system}=E_{bond}+E_{over}+E_{angle}+E_{tors}+E_{vdWaals}+E_{coulomb}+E_{Specific} \end{matrix} \end{aligned}$$

where $E_{bond}$ is the bond energy determined from the bond order. $E_{angle}$ and $E_{tors}$ the energies associated with the valence angle and torsional angle strains are the. The term $E_{over}$ is an energy penalty for the prevention of over-coordination of atoms (e.g. a penalty is applied if the carbon atoms make more than four bonds). The energy terms $E_{vdWaals}$ and $E_{Coulomb}$ present van der Waals interactions (dispersive contribution) and Coulomb (electrostatic contribution). The last term, $E_{Specific}$, represents other energy contributions specific to a system. They all have the same unit (kJ/mol)^5,37^.The energy of the system is calculated with the ReaxFF of ethanol and Al developed by Van Duin et al. ^37^, which is specifically developed for systems that include carbon, hydrogen, oxygen and Al^16^. This method is presented in the introduction section using Eqs. (1) and (2).

**Table 1:** The specification of the inputs for the nanoscale model. (**a**) The details of the geometry, Al slab and target molecules and environment conditions for the molecular dynamics simulation. Full details of the inputs, including the training and validation data set details, are provided in the supplementary notes S1. (**b**) The details of the MD code inputs and functions, including the periodic (*p*) and fixed (*f*) boundaries, pair style from the Reaxff for Hydrogen (H), Carbon (C), Oxygen (O) and Al, neighbour parameters that affect the building of pairwise neighbours list and energy minimisation style and value.

Sign	Description	Value
(a) MD simulation parameters		
$H_{sb}$	Height of the simulation box (m)	$4.2 \times 10^{-9}$
$W_{sb}$	Width of the simulation box (m)	$3.5 \times 10^{-9}$
$D_{sb}$	Depth of the simulation box (m)	$3.4 \times 10^{-9}$
$T_{Al}$	Thickness of the Aluminium slab (m)	$1.65 \times 10^{-9}$
$T_{O_E}$	Stopping tolerance for energy (–)	$10^{-10}$
$T_{O_F}$	Stopping tolerance for force (Kcal/mole-Angstroms)	$10^{-10}$
$V_{tm}$	Velocity of the targeted molecules (m/s)	$10^{-3}-1$
$T_{md}$	The environment temperature (K)	200–500
$N_{tm}$	Number of the targeted molecules Cases G (10), F (25), A (50), B (100), C (150) and D (200)	10–200

Function	Input	
(b) MD code input and functions		
Units	Real	
Dimension	3	
Boundary	*ppf*	
Pair style	Reax	
Pair coeff.	Reaxff H C O Al	
Neighbour	1.0 bin, every 20	
Thermo style	Costum, 10	
Time step	0.25 fs	
Minimisation	Conjugate gradient	
Style and value	$10^{-10}$	

MD method only considers the interactions with atoms in a spherical cut-off, which gives the linear scaling of the number of atoms^39^. In the next step, energy minimisation is carried out at the beginning of the simulation by using the Conjugate gradient algorithm  ^40^. For temperature control of the system, the Berendsen thermostat method  ^41^ is used, which resets the temperature of atoms and re-scales their velocity every time step. Since the temperature through this reaction is lower than 500 *K*, a time step of 0.5 *fs* is considered. The steady-state condition indicators in this stage are the total potential energy and pressure. Algorithm 1 presents the main structure of this stage, and Table 1a, b provide the detailed information of the MD simulations parameters and code imputs.

**Algorithm 1:**
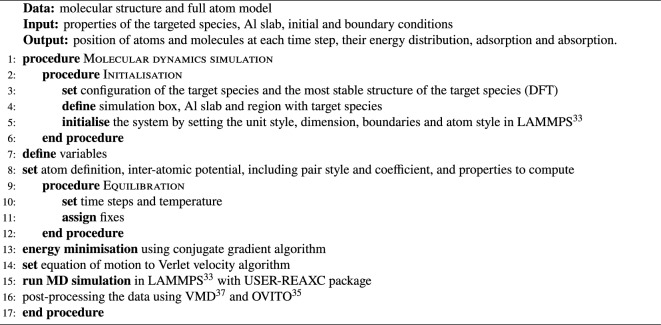
The algorithm for the molecular dynamics simulation (all-atom) code. We used a reactive force field (ReaxFF) alongside the large-scale atomic/molecular massively parallel simulator (LAMMPS^34^).

### Stage 2: training regression ML models for inferring the number of adsorbed molecules

In the first stage of our project, we conducted molecular dynamics simulations, which produced a set of training data $\textit{D}$ of *n* observations, $\textit{D}=\{(X_i,y_i) \mid i=1,\ldots ,n\}=(\textit{X},y)$, where *x* denotes the input conditions, such as the concentration, temperature, time, pressure and velocity, *y* denotes the number of adsorbed ethanol molecules, and $\textit{X}$ is the design matrix. To identify the important predictors while training the ML models we used the minimum redundancy maximum relevance (MRMR) Algorithm^42^, details of these features with their MRMR score are summarised in Table 2a. Concentration and pressure seem to be the most important features.

In the second stage, we used $\textit{D}$ to infer the relationship between the inputs and the number of adsorbed molecules using 28 parametric and non-parametric methods. For both methods, we used Bayesian analysis to find the probability of the parameters of the model ($\theta$) after the data observation  ^28^.

In parametric methods, we define a model that depends on some parameters ($\theta$), and then we determine the best value for the parameters using the Maximum a posteriori (MAP). Linear, robust and stepwise linear regressions are the parametric methods used in this work. Linear Regression model multiplies each predictor by a coefficient and sums them together to predict the response (Eq. 4)^28^.

4 $$\begin{aligned} f(x)=x^\top {\textbf {w}}, \quad y=f(x)+\varepsilon , \quad \varepsilon \sim \mathcal {N}(0,\sigma ^2_n), \quad {\textbf {w}} \sim \mathcal {N}(0,\Sigma _p) \end{aligned}$$

where *f* is the function of value, ${\textbf {w}}$ is the weight vector of linear model parameters, *n* is the number of observations, and *x* is the input vector. The observed values *y* differ from the function values by additive noise $\varepsilon$, which is a Gaussian distribution with variance $\sigma ^2_n$ and zero mean. We added a normal prior with covariance matrix $\Sigma _p$ to the weights to change and improve the usual least square problem.

5 $$\begin{aligned} p(y\mid \textit{X},{\textbf {w}})=\prod ^n_{i=1}p(y_i\mid \textit{X}_i,{\textbf {w}})=\prod ^n_{i=1}\frac{1}{\sqrt{2\pi \sigma _n}}exp\Bigg (-\frac{(y_i-x^\top _i{\textbf {w}})^2}{2\sigma ^2_n}\Bigg ) \sim \mathcal {N}(\textit{X}^\top {\textbf { w}}, \sigma ^2_n I) \end{aligned}$$

Then we used the Bayes’ rule to compute the posterior distribution as demonstrated in Eq. (6)^28^.

6 $$\begin{aligned} p({\textbf {w}}\mid \textit{X},y)=\frac{p(y\mid \textit{X},{\textbf {w}})p({\textbf {w}})}{p(y\mid \textit{X})} \sim \mathcal {N}\Bigg (\bar{{\textbf {w}}}=\frac{1}{\sigma ^2_n}A^{-1} \textit{X}y, A^{-1}\Bigg ), \quad A=\sigma ^2_n\textit{X} \textit{X}^\top + \Sigma _p^{-1} \end{aligned}$$

With training data being fixed, we need to find the value of ${\textbf {w}}$ at which the posterior distribution is maximum. Since the denominator of Eq. (6) (evidence) does not change with the weights. Hence, we only need to maximise the numerator. This principle is called maximum a posterior (MAP).

In non-parametric methods, the number of parameters depends on the dataset size, and the parameters are calculated with the help of Kernel functions. In this work, we used Gaussian process regression (GPR), support vector machine (SVM), and regression Tree, non-parametric methods. Gaussian processes (GP) is specified by mean (${{\,\mathrm{\mathbb {E}}\,}}f(x)=mx$) and covariance functions (*Cov*) in Eqs. (7) and (8).

7 $$\begin{aligned} & {{\,\mathrm{\mathbb {E}}\,}}[f(x)]=m(x), \quad Cov[f(x_1),f(x_2)]=k(x_1,x_2), \quad \forall n \in \mathbb {N}, \quad \forall x \in \mathbb {R}^d \end{aligned}$$

8 $$\begin{aligned} & \begin{pmatrix} f(x_1) \\ f(x_2) \\ ... \\ f(x_n)\end{pmatrix} \sim \mathcal {N} \begin{pmatrix} \begin{pmatrix} m(x_1) \\ m(x_2) \\ ... \\ m(x_n)\end{pmatrix}, \begin{pmatrix} k(x_1, x_1) & k(x_1, x_2) & ... & k(x_1, x_n)\\ k(x_2, x_1) & k(x_2, x_2) & ... & k(x_2, x_n) \\ . & . & . & . \\ k(x_n, x_1) & k(x_n, x_2) & ... & k(x_n, x_n)\end{pmatrix} \end{pmatrix} \sim \mathcal{G}\mathcal{P}(m(x), k(x,x')) \end{aligned}$$

The covariance matrix takes two points(e.g., $x_1$ and $x_2$) and returns the covariance between their value $f(x_1)$ and $f(x_2)$, so it will be equal to their kernel function $k(x_1,x_2)$, which depends on the posterior of these two points (Eq. 7). Different kernels were used in this work for training the GP, including squared exponential, Matern 5/2, exponential, and rational quadratic (Table 2). Kernel machines map the nonlinear data into a higher-dimensional space in nonlinear problems, reducing complexity and enhancing prediction. It is the same as linear regression when the kernel function is linear^43,44^.

where $\ell$ is the length scale, $\nu$ is the position parameter, $\alpha$ with $\ell$ as a scale mixture, $K_{\nu }$ is a modified Bessel function, and $\Gamma$ is the Gamma function^28,45^. With GPR, we can estimate the probability distribution of *f*(*x*), the prediction of new points, given all previous data, which will allow us to estimate the uncertainty of the predictions. SVM is another kernel machine with an $\epsilon$-insensitive error function. The MAP value of ${\textbf {w}}$ is calculated by minimising Eq. (9)^28^.

9 $$\begin{aligned} g_\epsilon (\textit{z})={\left\{ \begin{array}{ll}\mid \textit{z} \mid - \epsilon & if \mid \textit{z} \mid \ge \epsilon \\ 0 & otherwise.\end{array}\right. }, \quad min\Bigg (\frac{1}{2}\mid {\textbf {w}} \mid +C\sum ^n_{i=1}g_\epsilon (y_i-f_i)\Bigg ) \quad \,\xrightarrow []{Kernelised}\, \quad f(x_*)=\sum ^n_{i=1}\alpha _ik(x_i,x_*) \end{aligned}$$

where $\textit{z}=y_if_i$, $\epsilon$ is a margin of tolerance with no penalty for errors, $g_\epsilon (\textit{z})$ is the error function, $x_i$ is the $i^{th}$ training input, $x_*$ is the test input, *k* is the kernel (covariance) function, the parameter $C>0$ specifies the relative importance of the two terms, and coefficients $\alpha$ are calculated with the quadratic programming (QP) optimisation problem (the objective function is quadratic). In this project, we used different kernels to calculate the solution, including linear, quadratic, cubic, coarse, medium and fine Gaussian, with kernel scales of 8.9, 2.2 and 0.56, respectively.

For the regression tree model, first, the input data $\textit{D}$ are inserted in the root node. Then, it goes from the root down to the leaf nodes, using binary conditions to group data with similar response variables. Each sequence of nodes is called a branch^46^. For training the model, it tries to find the best binary conditions that split the observations into two groups. The best split is determined using the mean squared error (MSE) of both groups. The predicted value would be the group mean. When the node can not split more, it is considered a leaf. Finer trees have more leaves, hence a complex predictive model.

Fine tree model would take more time for a large data set and features, and the process would become more complex^46^. In this work, we used fine, medium and coarse trees with minimum leaf sizes of 4, 12 and 36, respectively. ML models might have the same accuracy level, although their results might differ. Ensemble tree models (models 13 and 14) use the residuals from the tree models as a response variable to train an additional regression tree, which provides a new set of predictions and residuals. The process of generating decision trees in the Boosted tree model is repeated multiple times, resulting in a series of trees. The initial tree makes a prediction, and each subsequent tree refines that prediction (learning rate $\alpha =0.1$). The final prediction is the sum of all the results, which is more accurate than the initial prediction. In a bagged decision tree (model 14), different trees are trained simultaneously and independently with a random subset of available data as the training data^47^. This means that every tree has unique training data. The results are averaged together for a final prediction. We used 30 individual trees (learners $n_L=30$) to find the average $N_{ad}$ prediction in the ensemble models of this work.

**Table 2:** Details of the features and kernel functions used in the ML models and training (a) MRMR score, standard deviation ($\sigma$) and the average value ($\mu$) of features. (b) Kernel functions that are used for training the GP and their expression. These Kernel functions are Stationary and non-degenerate. A stationary covariance function is a function of $r=\mid {x-x'}\mid$, which can be represented as a Fourier transform. A degenerate kernel has a finite rank (finite number of non-zero eigenvalues)^28^.

Feature	MRMR	$\sigma$	$\mu$
(a) Features score and specifications			
$N_{tm}$	1.97	68.42	87.5
*P* (kpa)	0.4	1.01	-0.17
$T_{md} (K)$	0.16	70.66	318.18
$V_{tm} (m/s)$	0.06	3.38	0.93
Time (ps)	0.05	88.9	147.29

Covariance function	Expression		
(b) Kernel functions used for the GPR models			
Exponential	$exp(-\frac{r}{\ell })$		
Squared exponential	$exp(-\frac{r^2}{2\ell ^2})$		
Rational quadratic	$(1+\frac{r^2}{2\alpha \ell ^2})^{-\alpha }$		
Matern	$\frac{1}{2^{\upsilon -1}\Gamma (\upsilon )}(\frac{\sqrt{2\upsilon }}{\ell }r)^\upsilon K_\upsilon (\frac{\sqrt{2\upsilon }}{\ell }r)$		

To complete the ML benchmark, we included six neural network (NN) models, including narrow, medium, wide, Bilayered, Trilayered (models 19 to 23) and optimised NN (model 28). NN models are inspired by brain, consist of interconnected neurons in a layered structure and charactrised by the number of fully connected layers ($n_{l_c}$), the size of each layer ($l_1$, $l_2$ and $l_3$) and the regularisation strength ($\lambda$)^47^. Stage 2 is summarised in Algorithm 2, details of each model including the hyper parameters are provided in Table 3 and implemented using MATLAB^48^.

**Algorithm 2:**
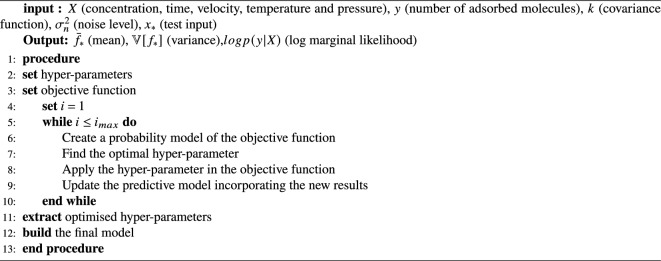
The algorithm for stage 2; the ML model training using

### Stage 3: testing and validation of the ML models

The last stage is validating and testing the prediction model on a new data set. It is important to avoid over-fitting while training the model with high accuracy; k-fold cross-validation is suggested for working on a small dataset (such as this work), with the number of values k lower than the number of instances. Hence five k folds would be sufficient for this work^50^. Cross-validation uses different portions of the data in the training process so that the model is not dependent on the specific portion of data. The model is trained with varying portions in each iteration. While holdout validations are more suitable for huge datasets^50^.

To ensure the robustness of our ML models, we utilised both validation methods, $25\%$ and $40\%$ holdout and 2-folds and 5-folds cross-validation. For all cases, we held $10\%$ of the data set separately, which is not seen by the validation and used it to see the performance of the final ML model. Additionally, we conducted five runs of the k-fold cross-validation, randomly selecting the five folds each time and comparing them to find the most suitable validation method for this dataset.

## Results and discussions

**Figure 1:**
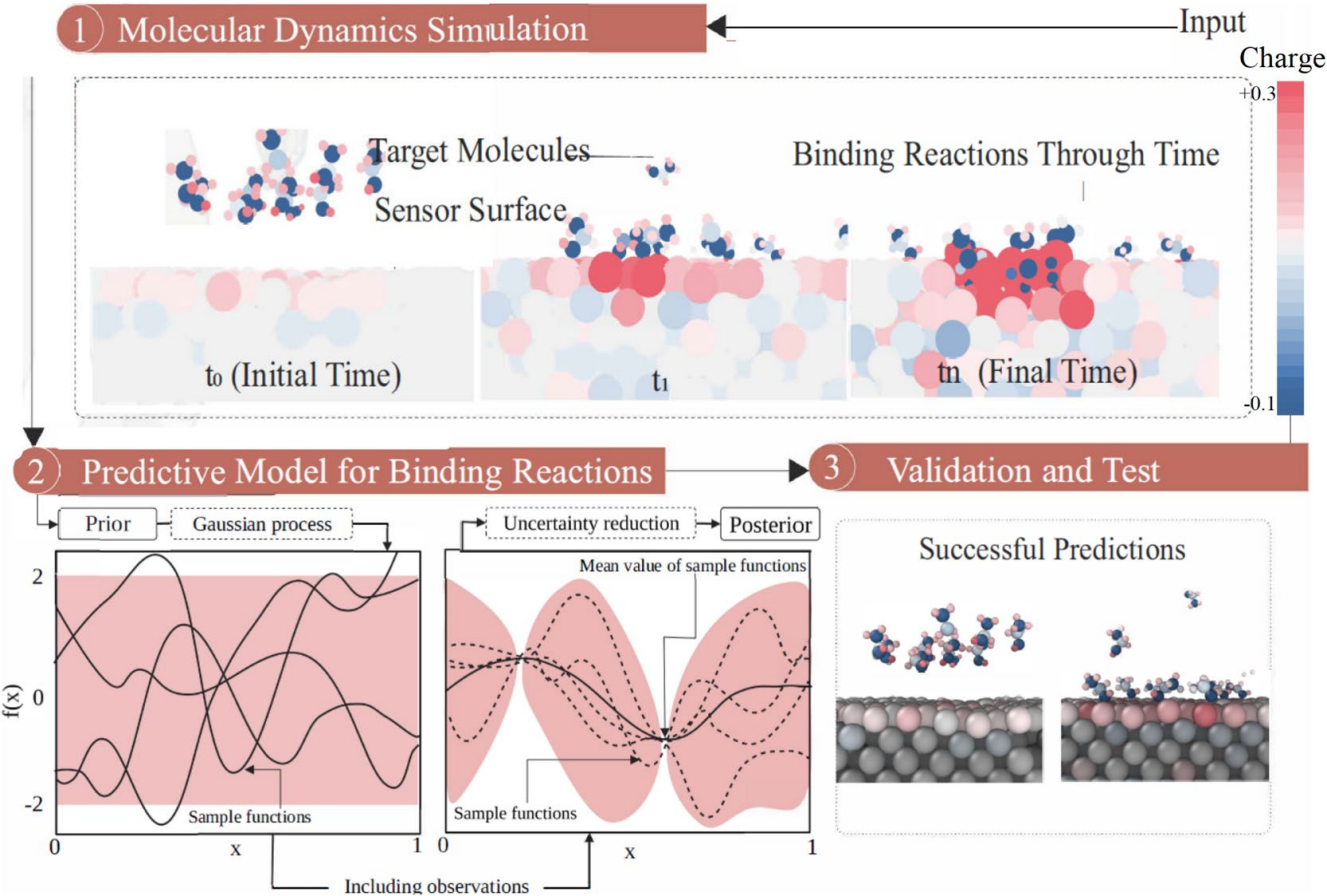
The three main steps of the current numerical framework for predicting the binding reactions: (1) molecular dynamics simulation, (2) binding prediction with the predictive model generated by the Bayesian ML method, and (3) test and validation. The prior and posterior for a Bayesian method for a simple regression problem are illustrated in step 2^51^, sample random Gaussian process functions are assigned to the sample input (*x*) and outputs (*f*(*x*)) and personalised based on the evidence in each loop.

In this section, we will first present the results obtained from the molecular dynamics simulations for ninety cases (Fig. 1-stage 1). Following that, we will evaluate the performance of 28 different ML models for inferring a relationship between the adsorption of the ethanol molecules on the Al slab for different velocities, concentrations and temperatures (Fig. 1-stages 2 and 3).

### MD simulation of ethanol molecule adsorption on an Al slab

In this part, the mechanism of ethanol adsorption on an Al slab in various conditions is studied using the results of the MD simulations. Due to the presence of Al in the propellant, this study covers a temperature range from 200 to 500 K. Table 1 provides the details of the simulation box, Al slab, concentration of ethanol molecules, velocity and temperature. The full details of the 90 different cases, convergence and results are provided in the supplementary materials S1 attached to this paper.

The simulation results provide the trajectory of the ethanol molecules on an Al slab through time, as presented in Fig. 1-stage 1 and coloured based on their charge. At the time $t_0$, the ethanol molecules were placed $5 \mathring{A}$ above the Al surface. First, the ethanol molecules get closer to the Al surface due to the electrostatic force between the hydroxyl group and the Al ($t_1$). The average adsorption distance is around $2.39 \mathring{A}$. These movements are driven explicitly by Oxygen and Al atom charges; the oxygen atom of the adsorbed ethanol has a negative charge ($-0.1$), while the Al atoms surrounding it have positive charges up to $+0.3$. The adsorption process completes at time $t_n$, which varies based on the concentration of the ethanol molecules, temperature and velocity. The steady-state condition indicators in this stage are the total potential energy and pressure.

Physical adsorption occurs in low temperatures (around 300*K*), and the oxygen of ethanol molecule gets close to the Al atom. However, with the increase in temperature, the adsorption becomes more complicated due to thermal vibrations of Al atoms and the decomposition of the ethanol molecule. Hence, in temperatures around 400 K and higher, the adsorption is a combination of physical and chemical interactions. These findings are aligned with the previous research on ethanol adsorption on Al^5^.

**Figure 2:**
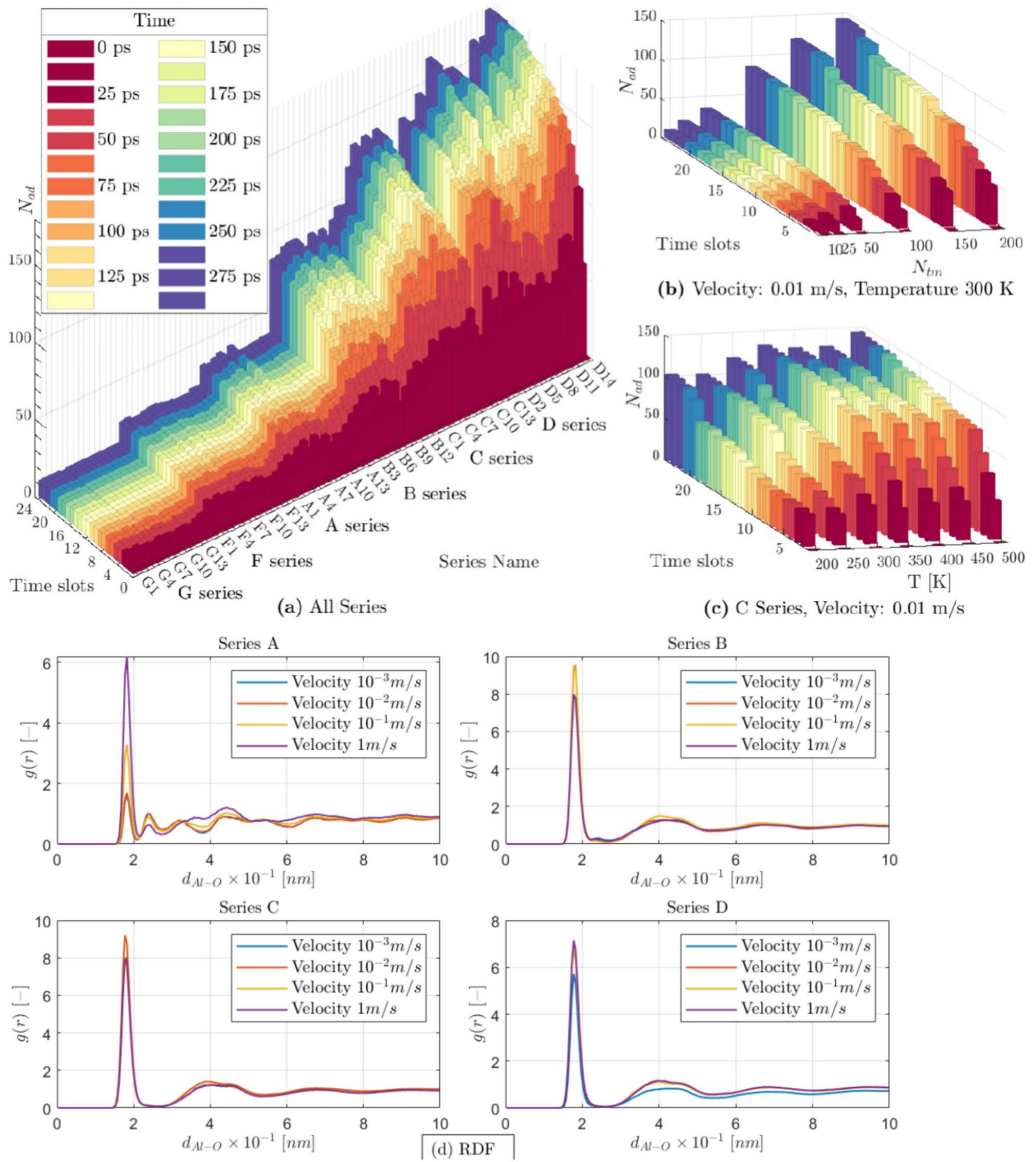
MD results (**a**) all series with a velocity of 0.01 m/s, (**b**) sample results for 0.01 m/s velocity and 300 k temperature, (**c**) C series with a velocity of 0.01 m/s and different temperature, (**d**) radial distribution function for four sample series in 300 K through time, in different velocities (from 0.001 to 1 m/s).

Figure 2a illustrates the number of adsorbed ethanol molecules ($N_{ad}$) through time for all 90 cases. The $N_{ad}$ is calculated based on the number of ethanol molecules within the adsorption distance. The $N_{ad}$ in time varies based on the concentration of the target molecules. In cases G and F, with the lowest concentration of target molecules, the ratio of adsorbed molecules over the total number of targets passing by the surface is close to one. At the same time, this ratio reduces as the concentration increases in cases A to D.

The fluctuations in $N_{ad}$ are due to two reasons: firstly, as more ethanol molecules are adsorbed, the concentration of the free ethanol molecule reduces, and they have more freedom to find free sites for adsorption; secondly, in higher temperatures, the vibration of atoms increases, and the Al surface gives more chance to the ethanol molecules for adsorption while making the adsorbed ethanol molecules fluctuate more.

With changes in temperature and velocity, adsorption shows a different behaviour for each case. Figure 2b presents changes of $N_{ad}$ through time with concentration for five different series with the same initial temperature and velocity; flow velocity of 0.01 m/s and temperature of 300 K. This result supports the general statement that increasing the concentration increases adsorption. The adsorption is completed after 2 to 3-time slots for the cases with low concentration, and it does not reach $100\%$ for high concentration cases, even if a significant time passes.

Figure 2c illustrates the effect of temperature on the adsorption of molecules for series C number of ethanol molecules $N_{tm}$ of 150). The variation of adsorption through time is close to an exponential curve for lower temperatures, and it gradually changes to a logarithmic curve as the concentration increases. To evaluate the slab properties and adsorption, the radial distribution function (RDF) is used (Eq. 10)  ^52^. It calculates the probability of finding a particle (in this case, Oxygen) at a specific distance from another particle (Al).

10 $$\begin{aligned} g(r)=c_{Al}^2 g_{Al-Al}(r)+2 c_O c_{Al}g_{Al-O}(r)+ c_O^2 g_{O-O}(r) \end{aligned}$$

where $c_{Al}$ and $c_{O}$ denote the concentration of Al and oxygen, $g_{Al-Al}$, $g_{Al-O}$ and $g_{O-O}$ are the three partials functions, which add up to the total RDF (*g*). Factor two appears as the $g_{Al-O}$ and $g_{O-Al}$ are identical^52^. Due to the high number of cases, only sixteen are presented in the RDF graph (Fig. 2d). The location of the radial distribution function peak presents the binding distance, which is the same for all cases ($1.8 \times 10^{10}$ m). The radial distribution function also varies with velocity but does not follow a specific pattern with a change in concentration.

The binding process does not follow a standard function of time, temperature or concentration, and it becomes more complicated with the increase in concentration. Another issue is the simulation time; on 16 nodes of a high-performance computing system, it usually takes 11–14 h for each case, while it increases by $25\%$ for series C and D with the highest concentrations. These two challenges in choosing a proper estimator and reducing the prediction time lead us to the next stage of the numerical framework, finding the relations using the regression ML methods.

### Evaluation of the regression ML methods for the adsorption behaviour

ML is an iterative process, and it is not always possible to choose which regression model would be the best choice for complex processes, such as binding reactions. Hence, it is important to evaluate them. In this part, we used the MD simulation results for training, testing and validating 28 ML regression models. The $N_{ad}$ is selected as the response variable, and velocity, concentration, temperature and time are set as the predictor variables for the training process. In order to assess the performance of these ML models and make a comparison, different metrics are considered, including the mean absolute error (MAE), root mean squared (RMSE), correlation coefficient (RSQ), as introduced in Eq. (11)^47^, and prediction time (PS).

11 $$\begin{aligned} MSE=\frac{1}{n}\sum _{i=1}^{n}(y_i-\widehat{y_i})^2, \quad RMSE=\sqrt{\frac{1}{n}\sum _{i=1}^{n}(y_i-\widehat{y_i})^2}, \quad RSQ=1-\frac{\frac{1}{n}\sum _{i=1}^{n}(y_i-\widehat{y_i})^2}{\frac{1}{n}\sum _{i=1}^{n}(y_i-\bar{y})^2}, \quad i=1,2,\ldots ,n \end{aligned}$$

where *n* is the number of observations, $y_i$ is the actual data points and $\widehat{y_i}$ is the predicted values, $y_i-\widehat{y_i}$ is the residual, and $\bar{y}$ is the the mean response value. Residuals close to zero indicate a good predictive model. The MSE value emphasises the large errors. Hence, low MSE indicates relatively few large errors in the predictions. RMSE has the same unit as the response value ($N_{ad}$), which means RMSE with a value of 4 means that the predictive $N_{ad}$ calculations are off by four adsorbed molecules on average. Both MSE and RMSE are good for comparing the models. However, they are not effective in determining if the model is objectively a good fit for the data. The RSQ value is suitable for comparing the model with a simple baseline model; for that, we consider a simple horizontal line that passes the mean response value ($\bar{y}$). If the predictions are accurate, the RSQ is close to one.

**Table 3:**
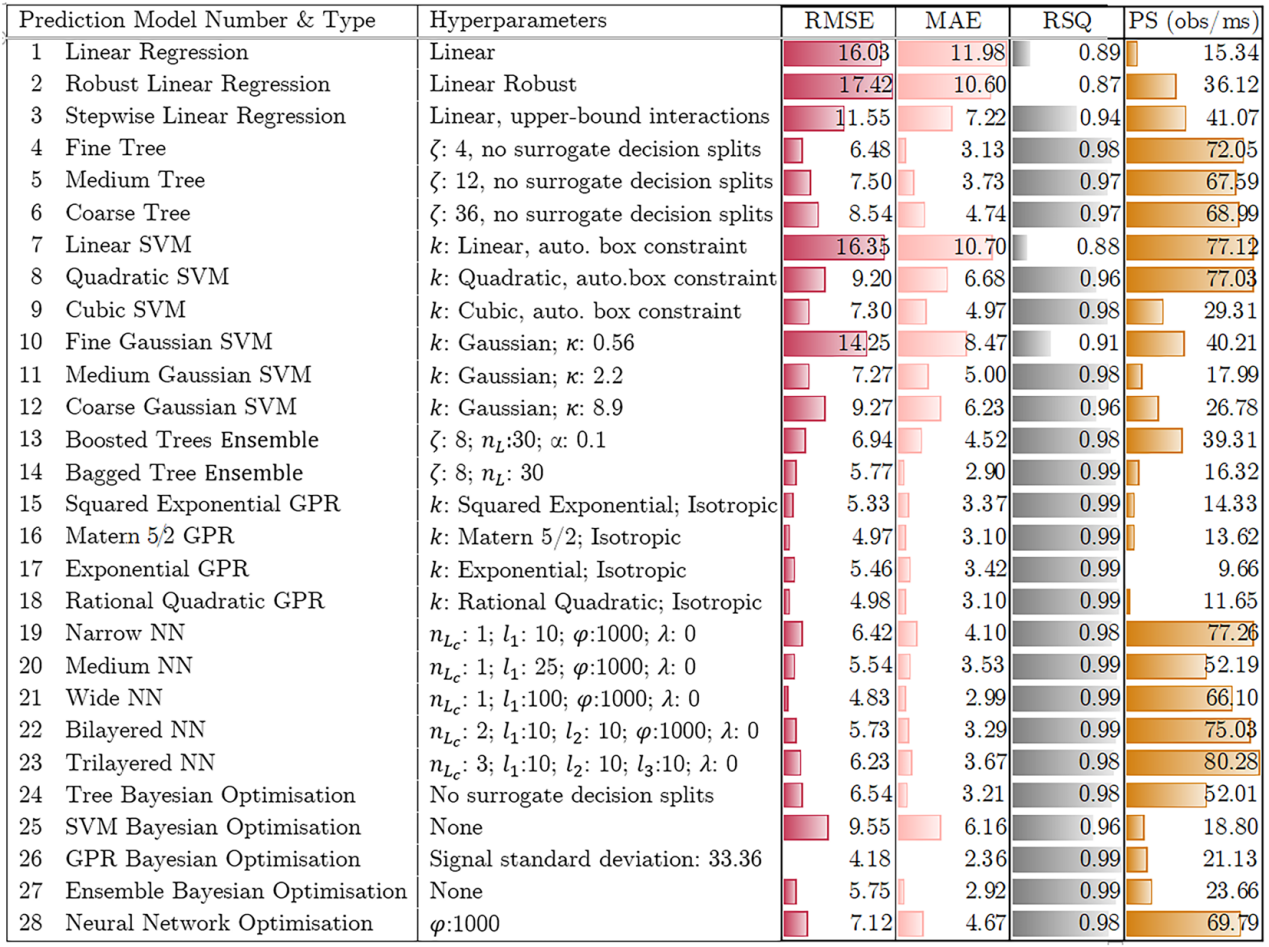
Details of the predictive models for a single run test data: training time, RMSE (root mean squared error), MAE (mean absolute error), and RSQ (correlation coefficient) for the test set.

Kernel function (*k*), Kernel scale ($\kappa$), minimum leaf size ($\zeta$) iteration limit ($\varphi$), number of learners ($n_l$), connected layers ($n_{l_c}$), layer size (*l*), regularisation strength ($\lambda$), learning rate ($\alpha$) and prediction speed (*PS*) are provided for related models (full details are available in the supplementary notes S1).

**Figure 3:**
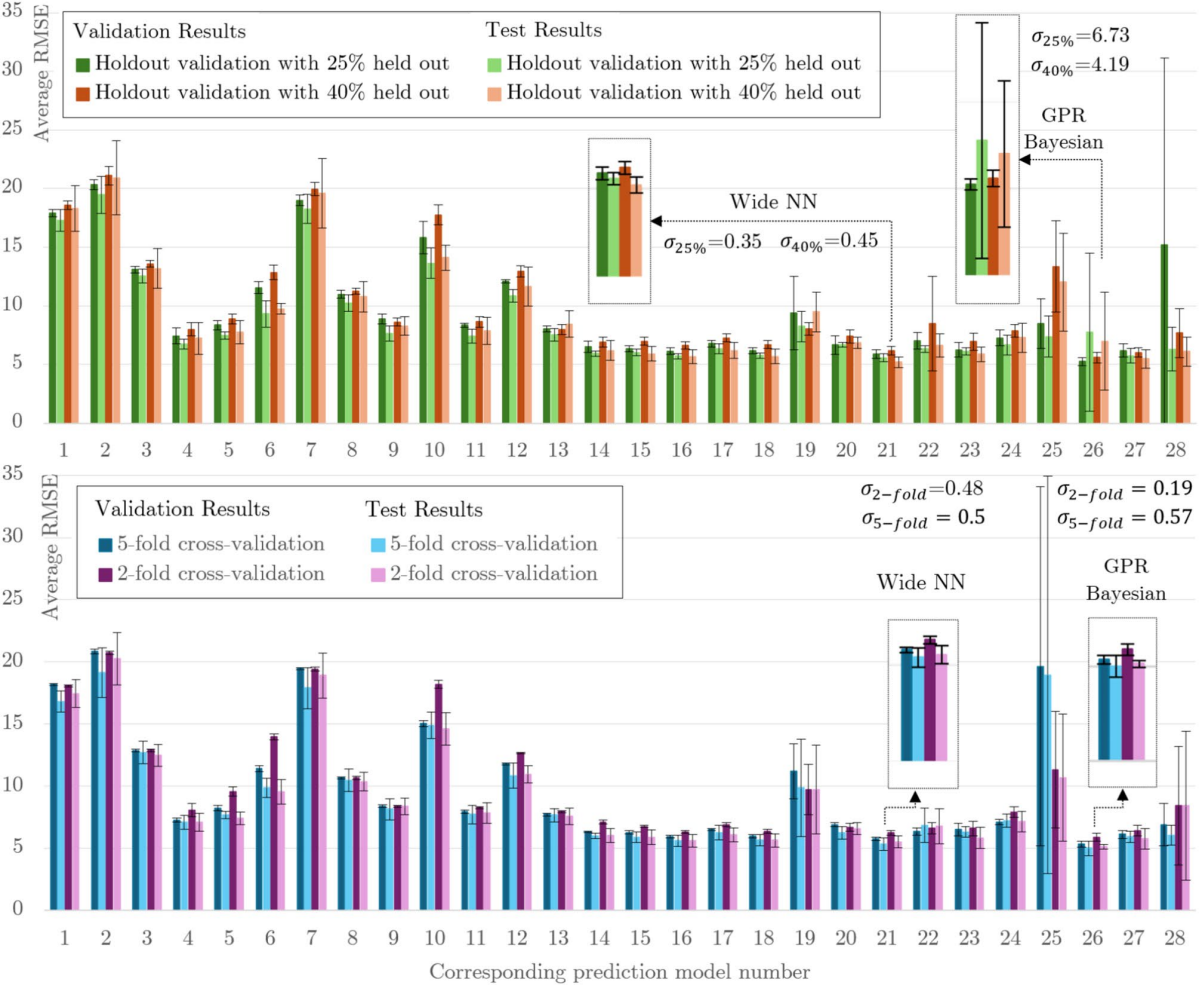
The statistical analysis of different ML models for two validation methods: holdout validation for 25% and 40% holdout and cross-validation for 2-folds and 5-folds. Both validation and test results are averages of five different runs, the error bars are calculated using the standard deviation ($\sigma$) of the five runs for each cases. The zoomed view of top two (lowest RMSE) prediction models, number 21 (wide NN) and number 26 (GPR Bayesian Optimisation) are provided. .

Figure 3 presents the validation and test results for the data set that has not been seen during the validation process. 5-fold cross validation method shows a lower standard deviation for all ML models except model 25 (SVM Optimisation). Model 25 already shows a how RMSE and is not suitable for our data set. Hence , cross-validation^49^ of data with five folds is used for this research while keeping $10\%$ of data for testing the prediction model.

Table 3 presents the prediction performance of 28 ML models for the test data. These test data were not used in the training and validation process. Models 1–3 in Table 3 are linear regression models, which are generally fast and easy to implement. However, they are not sufficient for predicting complex behaviour such as the adsorption process^53^. This point is evident in these results; due to the nonlinear nature of the training data, linear models, including linear regression (models 1–3) and linear SVM (model 7), failed to provide a high-accuracy ML model and have the highest MAE and the lowest RSQ. Their RMSE is up to 17.42, which means their prediction of the number of adsorbed molecules is off by 17. To avoid under-fitting resulting from the linear models, we applied more complex models. However, using complex models may take longer training times and may even capture noises, which could result in over-fitting. Over-fitted models may exhibit low error rates for the training data, but when tested on new data, they may show high error rates. Therefore, it is essential to choose a complex model that is flexible enough to accurately predict new data.

Models 4–6 are the fine, medium and coarse regression trees with minimum leaf size $\zeta$ of 4, 12 and 36, respectively. These trees differ based on the minimum number of observations allowed in a single leaf. As a result, a fine tree will have more leaves in total, which leads to more complex predictive models and an increase in training time. This point is also evident in the results, as presented in Table 3, *PS* increases from 40 obs/ms for linear models up to 72 obs/ms for the Tree models. They have a low range of MAE 3.13–4.74 but not an adequate RSQ (0.97–0.98). Nonlinear SVM models (models 8–12) can also achieve a comparably low MAE, but they still have a low RSQ. The ensemble models (models 13 and 14) consider the results from multiple models instead of relying on just one, which makes them more reliable. The best-performing tree model is the fine tree with a minimum leaf size of 4, with around 6.5 $N_{ad}$ overestimation; with the bagged tree ensemble model, the RMSE is reduced by 1. The results of the GPR models with different kernel functions (models 15–18) are presented in Table 3; based on the results, the optimisable GPR and MatÃ©rn 5/2 kernel function seem to give a more accurate model for the number of adsorption of target molecules on the Al slab. These models have the lowest RMSE, maximum RSQ, and a comparably low prediction speed. As predicted, as we are not dealing with a large dataset, increase in the connected layers for the NN models (models 19–23) shows an increase in the RMSE and PS and increase in the layer size, decreases the prediction error. Wide NN model with 100 number of neurons, has the lowest MAE and RMSE, but the prediction time is more than three times higher than the GPR model.

**Figure 4:**
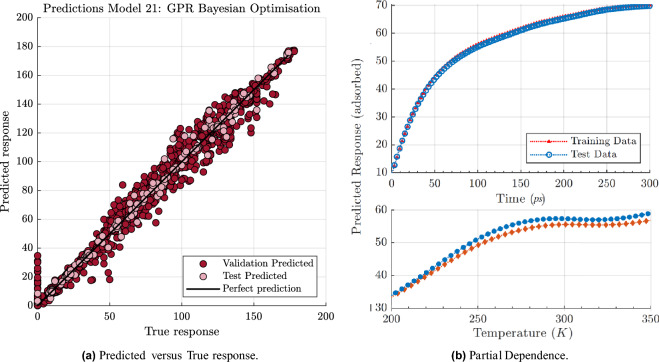
Training and validation of the Bayesian-based GPR optimised predictive model; (**a**) predicted versus true responses plot for validation and test data using the GPR with Bayesian optimisation model (predicted versus true responses for other models are available in the supplementary notes S1), (**b**) partial dependence of the predicted response of the adsorbed target molecules to time and temperature.

Bayesian optimisation is applied to find the best hyper-parameter combination, according to the performance of the previous combinations. Each model and the results for optimised models are presented in Table 3 models 24–28. Hyper-parameters are set before training the data; hence, it is important to choose the best one. The Bayesian optimisation helped to reduce the error by at least estimating one less wrong adsorbed molecule. The optimisation algorithm reduces the speed. The full details of validation and testing results, performance metrics, and model specification options for the 28 models are available in the supplementary notes S1. Here, we present the training and validation results for one example (GPR Bayesian optimisation, model 21) in Fig. 4. Figure 4a presents the predicted versus the actual values, which is helpful to evaluate whether any $N_{ad}$ is poorly modelled. Both axes present the $N_{ad}$, the *x* axis represents the true $N_{ad}$ value from the MD simulations data set, and the *y* axis represents the predicted $N_{ad}$ values from the GPR Bayesian model. The black line represents the perfect prediction, and the vertical distance from this line is the residual for each data point. To evaluate the model residuals depend on the predictor variables, we can have a look at Fig. 4b , which presents the partial dependence of the predicted response of the adsorbed target molecules to time and temperature. The results show a good prediction through time, although we have a slight overestimation through temperature.

The results show a good alignment between the predicted and the actual adsorbed ethanol everywhere, but in the lower left corner at the zero value. The wrong predicted values tend to be higher than the perfect prediction line, which means the model tends to overestimate the number of adsorbed molecules. If we have a look back at Fig. 2a at stage zero, the beginning point of the simulations that the ethanol molecules are set as a specific position in the simulation box, $N_{ad}$ for cases with high concentrations is high, while for cases with low concentration is zero. This is purely due to the fact that with high concentrations (cases A–D), ethanol molecules are already very close to the surface, and the adsorption is already happening. Hence, the outline data points on the validation data set are mainly dedicated to the cases with high concentrations. This observation indicates that the leading cause of the error for the ML model might have been the zero point. After stage zero, all cases start to show an increase in the adsorbed ethanol, which is captured well with the prediction model.

## Conclusion

This research demonstrates the use of machine learning and molecular dynamics simulations to enhance our capabilities in predicting the binding reactions at the nanoscale level. Advances in nanomaterials in recent years have significantly improved the development of high-performance devices. However, some deficiencies restrict their utilisation of sensitive devices. Processing materials at the nanoscale provides remarkable features, although they would trigger a series of problems; hence, detailed insight into changes in the nanoscale would be crucial. With this aim, we first used MD simulation to prepare a data set for the adsorption of ethanol on the Al surface using molecular dynamics simulations. We then evaluated different ML methods, such as linear regression, SVM, decision trees, ensemble, GPR, and optimised hyper-parameters with Bayesian optimisation, based on their capabilities in predicting the number of adsorption.

The MD simulation was applied for various conditions to study the effect of the concentration of targeted molecules, temperature and velocity on the thermodynamic properties, radial distribution function (RDF), and adsorption of ethanol. We used a large-scale atomic/molecular massively parallel simulator (LAMMPS) and the ReaxFF for these simulations. The results indicated the nonlinear nature of the adsorption process and its unique behaviour for different conditions. MD simulations also showed that an increase in velocity could significantly decrease the adsorption time and up to $80\%$ increase in adsorbed target molecules for the cases with low concentration.

However, there is a significant limitation in length and time using molecular dynamics simulations. ML assisted in tackling this challenge. With the data generated for ninety various molecular dynamics simulations , we trained, validated and tested 28 different ML models for prediction. As a result, the GPR model with Bayesian Optimisation of hyper-parameters and MatÃ©rn 5/2 kernel function showed a good prediction performance with the lowest RMSE and MAE and high RSQ (0.99) and comparably low prediction time (14–20 obs/s). Subsequently, the model was validated, and a successful prediction matched the available data. These ML models predict the number of adsorbed molecules off by 4–5 molecules. This number is not as effective in the high-concentration cases, but it shows the unsuitability of these predictive models for low-concentration models. With a closer look at the predicted data and comparison with the actual data, we found out the leading cause of prediction error is at stage zero when the simulation starts. The prediction models tend to overestimate the number of adsorbed molecules at that point. In future works, we can implement physic-based Bayesian ML models to improve the accuracy. This framework can be applied to other binding prediction tasks and complex target molecules.

## Supplementary Information

Supplementary Information.

## Data Availability

The entire data set is available in NDEx data repository http://www.ndexbio.org.

## References

[1] Kim, D., Kim, K., Kwon, G., Song, K. & Son, I. Self-propagating heat synthetic reactivity of fine aluminum particles via spontaneously coated nickel layer. *Sci. Rep.* **9**, 1033 (2019). 30705301 10.1038/s41598-018-36760-y PMC6355937

[2] Kim, D., Kim, K., Min, T., Kim, K. & Kim, S. Improved energetic-behaviors of spontaneously surface-mediated Al particles. *Sci. Rep.* **7**, 4659 (2017). 28680039 10.1038/s41598-017-04758-7 PMC5498582

[3] Liu, J., Liu, P. & Wang, M. Molecular dynamics simulations of aluminum nanoparticles adsorbed by ethanol molecules using the ReaxFF reactive force field. *Comput. Mater. Sci.* **151**, 95–105 (2018). 10.1016/j.commatsci.2018.04.054

[4] Gromov, A., Strokova, Y. & Teipel, U. Stabilization of metal nanoparticles-a chemical approach. *Chem. Eng. Technol. Ind. Chem. Plant Equip. Process Eng. Biotechnol.* **32**, 1049–1060 (2009).

[5] Liu, P., Liu, J. & Wang, M. Adsorption of ethanol molecules on the Al (1 1 1) surface: A molecular dynamic study. *R. Soc. Open Sci.* **6**, 181189 (2019). 30800368 10.1098/rsos.181189 PMC6366213

[6] Galvelis, R. *et al.* NNP/MM: Accelerating molecular dynamics simulations with machine learning potentials and molecular mechanics. *J. Chem. Inf. Model.* **63**, 5701–5708 (2023). 37694852 10.1021/acs.jcim.3c00773 PMC10577237

[7] Gu, S. *et al.* Others Can molecular dynamics simulations improve predictions of protein-ligand binding affinity with machine learning?. *Brief. Bioinform.* **24**, bbad008 (2023). 36681903 10.1093/bib/bbad008

[8] Nasr Esfahani, M. & Alaca, B. A review on size-dependent mechanical properties of nanowires. *Adv. Eng. Mater.* **21**, 1900192 (2019). 10.1002/adem.201900192

[9] Guida, F. *et al.* Peptide biosensors for anticancer drugs: Design in silico to work in denaturizing environment. *Biosens. Bioelectron.* **100**, 298–303 (2018). 28942212 10.1016/j.bios.2017.09.012

[10] Khoshbin, Z., Davoodian, N., Taghdisi, S. & Abnous, K. Metal organic frameworks as advanced functional materials for aptasensor design. *Spectrochim. Acta Part A Mol. Biomol. Spectrosc.* 121251 (2022). 10.1016/j.saa.2022.121251 35429856

[11] Abolhasan, R., Mehdizadeh, A., Rashidi, M., Aghebati-Maleki, L. & Yousefi, M. Application of hairpin DNA-based biosensors with various signal amplification strategies in clinical diagnosis. *Biosens. Bioelectron.* **129**, 164–174 (2019). 30708263 10.1016/j.bios.2019.01.008

[12] Akash, S., Bayıl, I., Hossain, M., Islam, M., Hosen, M., Mekonnen, A., Nafidi, H., Bin Jardan, Y., Bourhia, M. & Bin Emran, T. Novel computational and drug design strategies for inhibition of human papillomavirus-associated cervical cancer and DNA polymerase theta receptor by Apigenin derivatives. *Sci. Rep.* **13**, 16565 (2023). 10.1038/s41598-023-43175-x PMC10545697 37783745

[13] Dommer, A., Casalino, L., Kearns, F., Rosenfeld, M., Wauer, N., Ahn, S., Russo, J., Oliveira, S., Morris, C., Bogetti, A., et al. COVIDisAirborne: AI-enabled multiscale computational microscopy of delta SARS-CoV-2 in a respiratory aerosol. *Int. J. High Perform. Comput. Appl.* **37**, 28–44 (2023). 10.1177/10943420221128233 PMC9527558 36647365

[14] Polanski, J. 4.14 Chemoinformatics. SD Brown, R. Tauler, and BBT-CC Walczak (Eds.). pp. 459–506 (2009).

[15] Liu, P. *et al.* Molecular dynamic investigations of aluminum nanoparticles coated by the mixtures of ethanol and diethyl ether with different molecular proportions. *J. Nanopart. Res.* **22**, 1–14 (2020).35517915 10.1007/s11051-020-04974-9

[16] Hong, S. & Van Duin, A. Atomistic-scale analysis of carbon coating and its effect on the oxidation of aluminum nanoparticles by ReaxFF-molecular dynamics simulations. *J. Phys. Chem. C.* **120**, 9464–9474 (2016). 10.1021/acs.jpcc.6b00786

[17] Aktulga, H., Fogarty, J., Pandit, S. & Grama, A. Parallel reactive molecular dynamics: Numerical methods and algorithmic techniques. *Parallel Comput.* **38**, 245–259 (2012). 10.1016/j.parco.2011.08.005

[18] Van Duin, A., Verners, O. & Shin, Y. Reactive force fields: concepts of ReaxFF and applications to high-energy materials. *Int. J. Energ. Mater. Chem. Propulsion.* **12** (2013).

[19] Chenoweth, K., Van Duin, A. & Goddard, W. ReaxFF reactive force field for molecular dynamics simulations of hydrocarbon oxidation. *J. Phys. Chem. A* **112**, 1040–1053 (2008). 18197648 10.1021/jp709896w

[20] Noé, F., Tkatchenko, A., Müller, K. & Clementi, C. Machine learning for molecular simulation. *Annu. Rev. Phys. Chem.* **71**, 361–390 (2020). 32092281 10.1146/annurev-physchem-042018-052331

[21] Wang, Y., Ribeiro, J. & Tiwary, P. Machine learning approaches for analyzing and enhancing molecular dynamics simulations. *Curr. Opin. Struct. Biol.* **61**, 139–145 (2020). 31972477 10.1016/j.sbi.2019.12.016

[22] Alber, M. et al. & Others Integrating machine learning and multiscale modeling-perspectives, challenges, and opportunities in the biological, biomedical, and behavioral sciences. *NPJ Digit. Med.* **2**, 115 (2019). 10.1038/s41746-019-0193-y PMC6877584 31799423

[23] Weiel, M. *et al.* Dynamic particle swarm optimization of biomolecular simulation parameters with flexible objective functions. *Nat. Mach. Intell.* **3**, 727–734 (2021). 10.1038/s42256-021-00366-3

[24] Pandey, M. *et al.* The transformational role of GPU computing and deep learning in drug discovery. *Nat. Mach. Intell.* **4**, 211–221 (2022). 10.1038/s42256-022-00463-x

[25] Chu, Y., Zhang, Y., Wang, Q., Zhang, L., Wang, X., Wang, Y., Salahub, D., Xu, Q., Wang, J., Jiang, X., et al. A transformer-based model to predict peptide–HLA class I binding and optimize mutated peptides for vaccine design. *Nat. Mach. Intell.* **4**, 300–311 (2022).

[26] Unanue, E. From antigen processing to peptide-MHC binding. *Nat. Immunol.* **7**, 1277–1279 (2006). 17110945 10.1038/ni1206-1277

[27] Reynisson, B., Alvarez, B., Paul, S., Peters, B. & Nielsen, M. NetMHCpan-4.1 and NetMHCIIpan-4.0: improved predictions of MHC antigen presentation by concurrent motif deconvolution and integration of MS MHC eluted ligand data. *Nucleic Acids Res.* **48**, W449–W454 (2020). 10.1093/nar/gkaa379 PMC7319546 32406916

[28] Williams, C. & Rasmussen, C. *Gaussian processes for machine learning* 1–128 (MIT press Cambridge, MA, 2006).

[29] Savage, L. The foundations of statistics. 44–124 (Courier Corporation, 1972).

[30] Libbrecht, M. & Noble, W. Machine learning applications in genetics and genomics. *Nat. Rev. Genet.* **16**, 321–332 (2015). 25948244 10.1038/nrg3920 PMC5204302

[31] Lewis, P. A likelihood approach to estimating phylogeny from discrete morphological character data. *Syst. Biol.* **50**, 913–925 (2001). 12116640 10.1080/106351501753462876

[32] Shahbazi, F., Esfahani, M., Jabbari, M. & Keshmiri, A. A Molecular Dynamics Model for Biomedical Sensor Evaluation Nanoscale Numerical Simulation of an Aluminum-Based Biosensor. In *2022 44th Annual International Conference Of The IEEE Engineering In Medicine and Biology Society (EMBC)* pp. 613–616 (2022). 10.1109/EMBC48229.2022.9871498 36086108

[33] Shahbazi, F., Jabbari, M., Esfahani, M. & Keshmiri, A. Microfluidic-Integrated Biosensors. Applied Complex Flow: Applications Of Complex Flows And CFD. pp. 21–42 (2023)

[34] Thompson, A. *et al.* LAMMPS - a flexible simulation tool for particle-based materials modeling at the atomic, meso, and continuum scales. *Comp. Phys. Comm.* **271**, 108171 (2022). 10.1016/j.cpc.2021.108171

[35] Iype, E., Khalfay, Z., Chaudhuri, R. & Kumar, B. Epsomite dehydration: A molecular dynamics study. *J. Energy Stor.* **20**, 337–343 (2018). 10.1016/j.est.2018.10.005

[36] Stukowski, A. Ovito open visualization tool (2015).

[37] Senftle, T., Hong, S., Islam, M., Kylasa, S., Zheng, Y., Shin, Y., Junkermeier, C., Engel-Herbert, R., Janik, M., Aktulga, H. & Others The ReaxFF reactive force-field: development, applications and future directions. *Npj Comput. Mater.* **2**, 1–14 (2016).

[38] Humphrey, W., Dalke, A. & Schulten, K. VMD: visual molecular dynamics. *J. Mol. Graph.* **14**, 33–38 (1996). 8744570 10.1016/0263-7855(96)00018-5

[39] Li, Y., Xu, J. & Li, D. Molecular dynamics simulation of nanoscale liquid flows. *Microfluid. Nanofluid.* **9**, 1011–1031 (2010). 10.1007/s10404-010-0612-5

[40] Guénolé, J. *et al.* Assessment and optimization of the fast inertial relaxation engine (fire) for energy minimization in atomistic simulations and its implementation in lammps. *Comput. Mater. Sci.* **175**, 109584 (2020). 10.1016/j.commatsci.2020.109584

[41] Berendsen, H., Postma, J. . Di., Nola, A. & van,. Gunsteren, WF and Haak. *JR. J. Chem. Phys.* **81**, 3684 (1984).

[42] Ding, C. & Peng, H. Minimum redundancy feature selection from microarray gene expression data. *J. Bioinf. Comput. Biol.* **3**, 185–205 (2005). 10.1142/S0219720005001004 15852500

[43] Chen, P., Fan, R. & Lin, C. A study on SMO-type decomposition methods for support vector machines. *IEEE Trans. Neural Netw.* **17**, 893–908 (2006). 16856653 10.1109/TNN.2006.875973

[44] Fan, R., Chen, P., Lin, C. & Joachims, T. Working set selection using second order information for training support vector machines. *J. Mach. Learn. Res.* **6** (2005).

[45] Abramowitz, M. & Stegun, I. With formulas, graphs, and mathematical tables. *Natl. Bureau Standards Appl. Math. Ser. E.* **55**, 953 (1965).

[46] Brieman, L., Friedman, J., Stone, C. & Olshen, R. Classification and regression tree analysis. (CRC Press: Boca Raton, FL, USA,1984).

[47] The MathWorks Inc. Statistics and machine learning toolbox. Statistics and machine learning toolbox documentation. (The MathWorks Inc., Natick, Massachusetts, United States, 2022), https://www.mathworks.com/help/stats/index.html.

[48] Inc., T. MATLAB version: 9.13.0 (R2022b). (The MathWorks Inc., 2022). https://www.mathworks.com

[49] King, R., Orhobor, O. & Taylor, C. Cross-validation is safe to use. *Nat. Mach. Intell.* **3**, 276–276 (2021). 10.1038/s42256-021-00332-z

[50] Yadav, S. & Shukla, S. Analysis of k-fold cross-validation over hold-out validation on colossal datasets for quality classification. In *2016 IEEE 6th International Conference On Advanced Computing (IACC)*, pp. 78–83 (2016).

[51] Rasmussen, C. Gaussian processes in machine learning. Summer School On Machine Learning. pp. 63–71 (2003).

[52] Stukowski, A. Visualization and analysis of atomistic simulation data with OVITO-the Open Visualization Tool. *Modell. Simul. Mater. Sci. Eng.* **18**, 015012 (2009). 10.1088/0965-0393/18/1/015012

[53] Abbasi, E., Moghaddam, M. & Kowsari, E. A systematic and critical review on development of machine learning based-ensemble models for prediction of adsorption process efficiency. *J. Clean. Prod.* **379**, 134588 (2022). 10.1016/j.jclepro.2022.134588

