# Supplementary material for: Assessment of machine learning models trained by molecular dynamics simulations results for inferring ethanol adsorption on an aluminium surface
Source: Sci Rep. 2024 Sep 3;14:20437. doi: 10.1038/s41598-024-71007-z (PMC11372171; doi:10.1038/s41598-024-71007-z)
Supplement: Supplementary file 1 — Supplementary Information. [file 41598_2024_71007_MOESM1_ESM.pdf]

Machine Learning-Aided Molecular Dynamics Simulation for Prediction of Binding Kinetics (Supplementary Notes)

Fatemeh Shahbazi[1,2,*], **Mohammad Nasr Esfahani**[3], **Amir Keshmiri**[2], **and Masoud Jabbari**[4]

[1]Warwick Manufacturing Group (WMG), University of Warwick, Coventry, CV4 7AL, UK
[2]Department of Fluids & Environment, School of Engineering, University of Manchester, M13 9QS, UK
[3]School of Physics, Engineering and Technology, University of York, York YO10 5DD, UK
[4]School of Mechanical Engineering, University of Leeds, Leeds, LS2 9JT, UK
[*]fatemeh.shahbazi@warwick.ac.uk

ABSTRACT

Chemical sensors provide new solutions to address some of the world's biggest challenges, including climate change, energy and healthcare. Understanding molecule binding kinetics and thermodynamics is essential in enhancing the design and functionality of chemical sensors. To contribute to this field, we have developed a numerical framework to predict the binding kinetics without requiring experimental inputs. Once the target molecules and fictional surface are identified, the details alongside the environment and mass transport are included as input to this code. The output would be the predictive model of target molecule behaviour passing by the surface. This framework comprises an all-atom molecular dynamics model and a Bayesian machine learning model for predicting affinity. Different predictive models have been trained, and the Bayesian-based Gaussian process regression (GPR) best predicts the binding reaction amongst them all. The predictive model is validated for an aluminium-based platform. The proposed numerical framework has the potential to be generalised and therefore, contribute to future low-cost binding reaction estimations, providing a valuable tool for industry and experimentalists.

List of Figures

1S Molecular dynamics simulation results for series A, B, C, D, F and G in different velocities . . . 5
2S Molecular dynamics simulation results for series A, B, C, D, F and G in different temperatures (200 to 500 K) . . 6
3S Adsorption results for the training cases in different velocities at 300 K . . . 7
4S The result of Linear prediction models; prediction response versus the true responses for Linear, Robust and Step-wise Linear Regression models. . . . 10
5S The result of Tree predictions models; prediction response versus the true responses for Fine, Medium, and Coarse Tree models. . . . 11
6S The results of SVM prediction models; prediction response versus the true responses for Linear, Quadratic and Cubic SVM models. . . . 12
7S The results of SVM Gaussian prediction models; prediction response versus the true responses for Fine, Medium and Coarse Gaussian SVM models. . . . 13
8S The results of Ensemble predictions models; prediction response versus the true responses for Boosted and Bagged Trees Ensemble models. . . . 14
9S The results of GPR and Ensemble predictions models; prediction response versus the true responses for Squared Exponential, Matern 5/2, Exponential and Rational Quadratic GPR models. . . . 15
10S The results of Bayesian optimisation based predictions models; for Trees, Ensemble, SVM and GPR. . . . 16

List of Tables

1S The specification of the inputs for the nanoscale model for series A - The details of the target molecules, velocity and temperature. . . . 2
2S The specification of the inputs for the nanoscale model for series B - The details of the target molecules, velocity and temperature . . . 2
3S The specification of the inputs for the nanoscale model for series C - The details of the target molecules, velocity and temperature . . . 3

4S The specification of the inputs for the nanoscale model for series D - The details of the target molecules, velocity and temperature . . . 3
5S The specification of the inputs for the nanoscale model for series F - The details of the target molecules, velocity and temperature . . . 3
6S The specification of the inputs for the nanoscale model for series G - The details of the target molecules, velocity and temperature . . . 4
7S Details of the predictive models; Hyperparameters. . . . 8
8S Details of the predictive models; training time, RMSE (root mean squared error), MSE (mean squared error), RSQ (correlation coefficient) and MAE (Mean absolute error). . . . 9
9S Details of the predictive models; prediction speed, training time and model size. . . . 9

Supplementary information

Table 1S. The specification of the inputs for the nanoscale model for series A - The details of the target molecules, velocity and temperature.

Case	Velocity [m/s]	Temperature [K]	Number of targets	Number of atoms
MD-A_01	1	300	50	2210
MD-A_02	0.1	300	50	2210
MD-A_03	0.01	300	50	2210
MD-A_04	0.001	300	50	2210
MD-A_05	0.0001	300	50	2210
MD-A_06	0.00001	300	50	2210
MD-A_07	0.000001	300	50	2210
MD-A_08	0.0000001	300	50	2210
MD-A_09	0.01	200	50	2210
MD-A_10	0.01	250	50	2210
MD-A_11	0.01	350	50	2210
MD-A_12	0.01	400	50	2210
MD-A_13	0.01	450	50	2210
MD-A_14	0.01	500	50	2210

Table 2S. The specification of the inputs for the nanoscale model for series B - The details of the target molecules, velocity and temperature

Case	Velocity [m/s]	Temperature [K]	Number of targets	Number of atoms
MD-B_01	1	300	100	2660
MD-B_02	0.1	300	100	2660
MD-B_03	0.01	300	100	2660
MD-B_04	0.001	300	100	2660
MD-B_05	0.0001	300	100	2660
MD-B_06	0.00001	300	100	2660
MD-B_07	0.000001	300	100	2660
MD-B_08	0.0000001	300	100	2660
MD-B_09	0.01	200	100	2660
MD-B_10	0.01	250	100	2660
MD-B_11	0.01	350	100	2660
MD-B_12	0.01	400	100	2660
MD-B_13	0.01	450	100	2660
MD-B_14	0.01	500	100	2660

Table 3S. The specification of the inputs for the nanoscale model for series C - The details of the target molecules, velocity and temperature

Case	Velocity [m/s]	Temperature [K]	Number of targets	Number of atoms
MD-C_01	1	300	150	3110
MD-C_02	0.1	300	150	3110
MD-C_03	0.01	300	150	3110
MD-C_04	0.001	300	150	3110
MD-C_05	0.0001	300	150	3110
MD-C_06	0.00001	300	150	3110
MD-C_07	0.000001	300	150	3110
MD-C_08	0.0000001	300	150	3110
MD-C_09	0.01	200	150	3110
MD-C_10	0.01	250	150	3110
MD-C_11	0.01	350	150	3110
MD-C_12	0.01	400	150	3110
MD-C_13	0.01	450	150	3110
MD-C_14	0.01	500	150	3110

Table 4S. The specification of the inputs for the nanoscale model for series D - The details of the target molecules, velocity and temperature

Case	Velocity [m/s]	Temperature [K]	Number of targets	Number of atoms
MD-D_01	1	300	200	3560
MD-D_02	0.1	300	200	3560
MD-D_03	0.01	300	200	3560
MD-D_04	0.001	300	200	3560
MD-D_05	0.0001	300	200	3560
MD-D_06	0.00001	300	200	3560
MD-D_07	0.000001	300	200	3560
MD-D_08	0.0000001	300	200	3560
MD-D_09	0.01	200	200	3560
MD-D_10	0.01	250	200	3560
MD-D_11	0.01	350	200	3560
MD-D_12	0.01	400	200	3560
MD-D_13	0.01	450	200	3560
MD-D_14	0.01	500	200	3560

Table 5S. The specification of the inputs for the nanoscale model for series F - The details of the target molecules, velocity and temperature

Case	Velocity [m/s]	Temperature [K]	Number of targets	Number of atoms
MD-F_01	20	300	25	1985
MD-F_02	10	300	25	1985
MD-F_03	5	300	25	1985
MD-F_04	1	300	25	1985
MD-F_05	0.5	300	25	1985
MD-F_06	0.4	300	25	1985
MD-F_07	0.3	300	25	1985
MD-F_08	0.2	300	25	1985
MD-F_09	0.1	300	25	1985
MD-F_10	0.05	300	25	1985
MD-F_11	0.01	300	25	1985
MD-F_12	0.01	200	25	1985

Table 5S continued from previous page				
MD-F_13	0.01	250	25	1985
MD-F_14	0.01	350	25	1985
MD-F_15	0.01	400	25	1985
MD-F_16	0.01	450	25	1985
MD-F_17	0.01	500	25	1985

Table 6S. The specification of the inputs for the nanoscale model for series G - The details of the target molecules, velocity and temperature

Case	Velocity [m/s]	Temperature [K]	Number of targets	Number of atoms
MD-G_01	20	300	10	1850
MD-G_02	10	300	10	1850
MD-G_03	5	300	10	1850
MD-G_04	1	300	10	1850
MD-G_05	0.5	300	10	1850
MD-G_06	0.4	300	10	1850
MD-G_07	0.3	300	10	1850
MD-G_08	0.2	300	10	1850
MD-G_09	0.1	300	10	1850
MD-G_10	0.05	300	10	1850
MD-G_11	0.01	300	10	1850
MD-G_12	0.01	200	10	1850
MD-G_13	0.01	250	10	1850
MD-G_14	0.01	350	10	1850
MD-G_15	0.01	400	10	1850
MD-G_16	0.01	450	10	1850
MD-G_17	0.01	500	10	1850

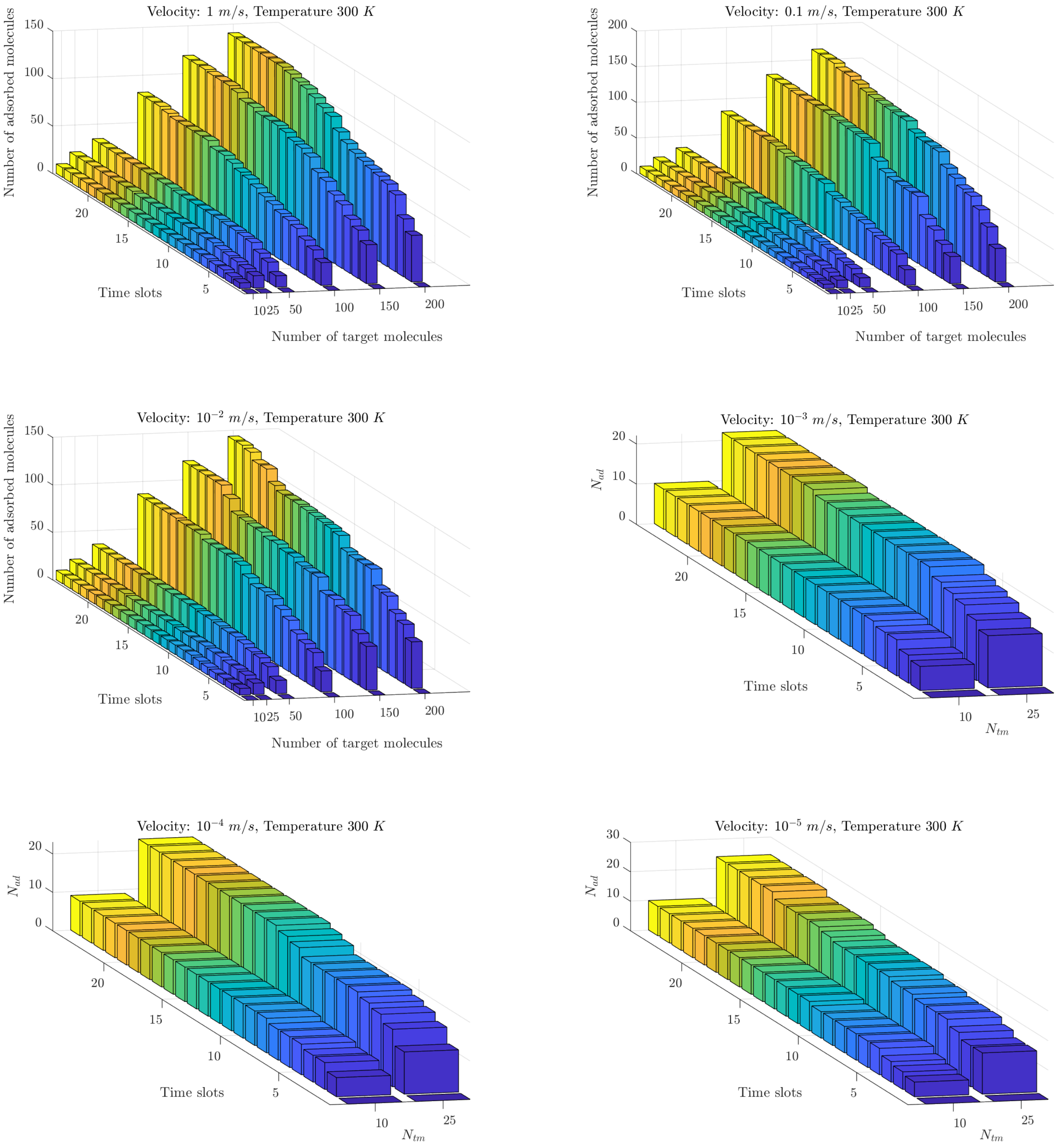

Figure 1S. Molecular dynamics simulation results for series A, B, C, D, F and G in different velocities

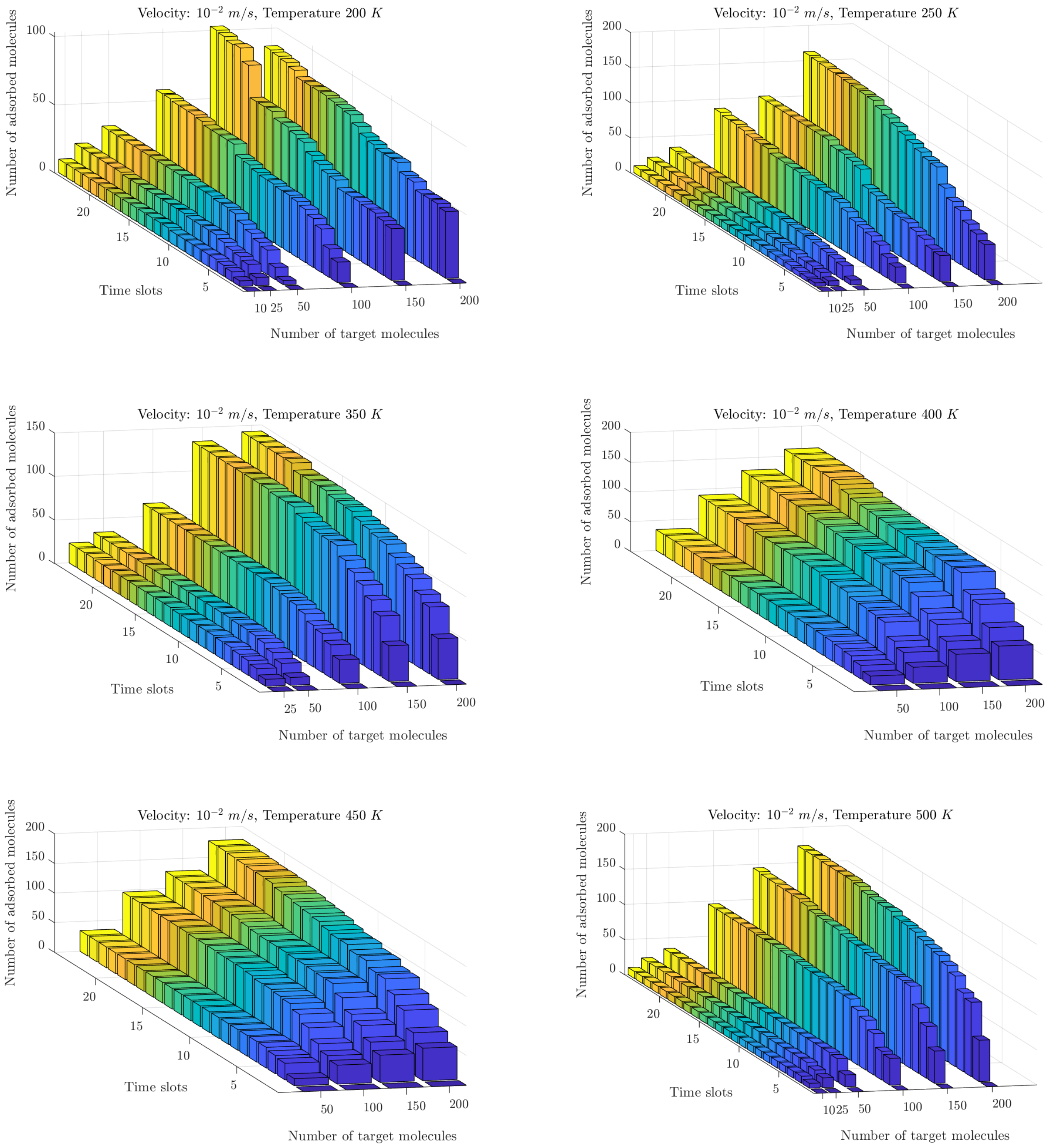

Figure 2S. Molecular dynamics simulation results for series A, B, C, D, F and G in different temperatures (200 to 500 K)

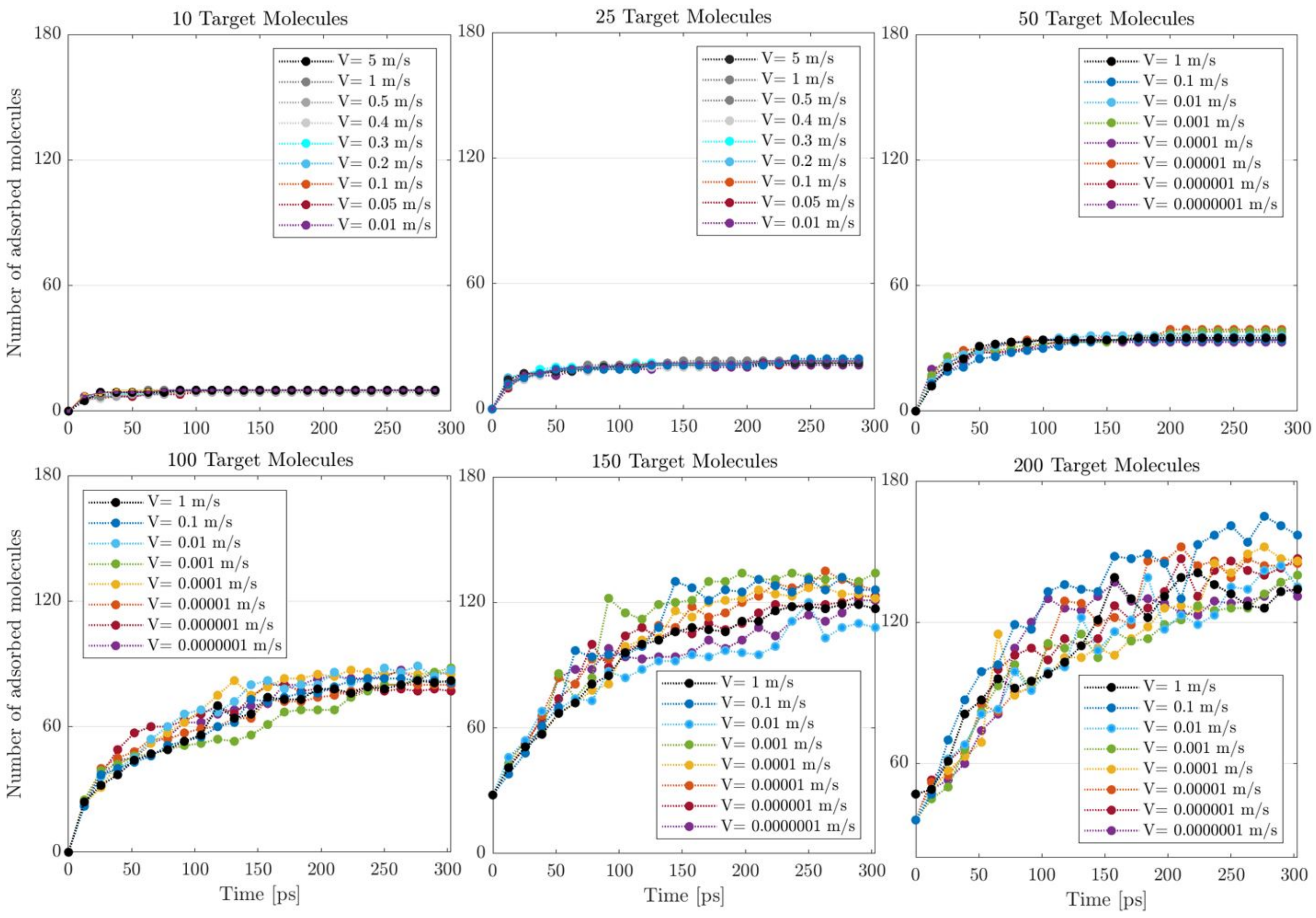

Figure 3S. Adsorption results for the training cases in different velocities at 300 K

Stage 2 results: developing predictive models

Table 7S. Details of the predictive models; Hyperparameters.

Prediction Model	Hyperparameters
1 Linear Regression	Terms: Linear; Robust option: Off
2 Robust Linear Regression	Terms: Linear; Robust option: On
3 Stepwise Linear Regression	Initial terms: Linear; Upper bound on terms: Interactions; Maximum number of steps: 1000
4 Fine Tree	Minimum leaf size: 4; Surrogate decision splits: Off
5 Medium Tree	Minimum leaf size: 12; Surrogate decision splits: Off
6 Coarse Tree	Minimum leaf size: 36; Surrogate decision splits: Off
7 Linear SVM	Kernel function: Linear; Kernel scale: Automatic; Box constraint: Automatic; Epsilon: Auto; Standardize data: Yes
8 Qudratic SVM	Kernel function: Quadratic; Kernel scale: Automatic; Box constraint: Automatic; Epsilon: Auto; Standardize data: Yes
9 Cubic SVM	Kernel function: Cubic; Kernel scale: Automatic; Box constraint: Automatic; Epsilon: Auto; Standardize data: Yes
10 Fine Gaussian SVM	Kernel function: Gaussian; Kernel scale: 0.56; Box constraint: Automatic; Epsilon: Auto; Standardize data: Yes
11 Medium Gaussian SVM	Kernel function: Gaussian; Kernel scale: 2.2; Box constraint: Automatic; Epsilon: Auto; Standardize data: Yes
12 Coarse Gaussian SVM	Kernel function: Gaussian; Kernel scale: 8.9; Box constraint: Automatic; Epsilon: Auto; Standardize data: Yes
13 Boosted Trees Ensemble	Minimum leaf size: 8; Number of learners: 30; Learning rate: 0.1; Number of predictors to sample: Select All
14 Bagged Trees Ensemble	Minimum leaf size: 8; Number of learners: 30; Number of predictors to sample: Select All
15 Squared Exponential GPR	Basis function: Constant; Kernel function: Squared Exponential; Use isotropic kernel: Yes; Kernel scale: Automatic; Signal standard deviation: Automatic; Sigma: Automatic; Standardize data: Yes; Optimize numeric parameters: Yes
16 Maten 5/2 GPR	Basis function: Constant; Kernel function: Matern 5/2; Use isotropic kernel: Yes; Kernel scale: Automatic; Signal standard deviation: Automatic; Sigma: Automatic; Standardize data: Yes; Optimize numeric parameters: Yes
17 Exponential GPR	Basis function: Constant; Kernel function: Exponential; Use isotropic kernel: Yes; Kernel scale: Automatic; Signal standard deviation: Automatic; Sigma: Automatic; Standardize data: Yes; Optimize numeric parameters: Yes
18 Rational Quadratic GPR	Basis function: Constant; Kernel function: Rational Quadratic; Use isotropic kernel: Yes; Kernel scale: Automatic; Signal standard deviation: Automatic; Sigma: Automatic; Standardize data: Yes; Optimize numeric parameters: Yes
19 Tree Bayesian	Surrogate decision splits: Off
20 SVM Bayesian	None
21 GPR Bayesian	Signal standard deviation: 33.3548; Optimize numeric parameters: Yes
22 Kernel Bayesian	Iteration limit: 1000
23 Ensemble Bayesian	None

Table 8S. Details of the predictive models; training time, RMSE (root mean squared error), MSE (mean squared error), RSQ (correlation coefficient) and MAE (Mean absolute error).

Prediction Model	RMSE		MAE		RSQ	
	Validation	Test	Validation	Test	Validation	Test
1 Linear Regression	18.26	16.03	13.68	11.98	0.85	0.89
2 Robust Linear Regression	20.9	17.42	12.81	10.6	0.8	0.87
3 Stepwise Linear Regression	13	11.55	8.16	7.22	0.92	0.94
4 Fine Tree	7.5	6.48	3.84	3.13	0.97	0.98
5 Medium Tree	8.51	7.5	4.75	3.73	0.97	0.97
6 Coarse Tree	11.39	8.54	6.67	4.74	0.94	0.97
7 Linear SVM	19.54	16.35	12.74	10.7	0.83	0.88
8 Qudratic SVM	10.77	9.2	7.32	6.68	0.95	0.96
9 Cubic SVM	8.46	7.3	5.68	4.97	0.97	0.98
10 Fine Gaussian SVM	15.1	14.25	9.59	8.47	0.9	0.91
11 Medium Gaussian SVM	7.92	7.27	5.41	5	0.97	0.98
12 Coarse Gaussian SVM	11.89	9.27	7.73	6.23	0.94	0.96
13 Boosted Trees Ensemble	7.89	6.94	5.14	4.52	0.97	0.98
14 Bagged Trees Ensemble	6.42	5.77	3.55	2.9	0.98	0.99
15 Squared Exponential GPR	6.42	5.33	4.08	3.37	0.98	0.99
16 Maten 5/2 GPR	6.11	4.97	3.75	3.1	0.98	0.99
17 Exponential GPR	6.65	5.46	3.91	3.42	0.98	0.99
18 Rational Quadratic GPR	6.15	4.98	3.75	3.1	0.98	0.99
19 Tree Bayesian	7.48	6.54	3.76	3.21	0.97	0.98
20 SVM Bayesian	11.33	9.55	7.12	6.16	0.94	0.96
21 GPR Bayesian	5.49	4.18	3.15	2.36	0.99	0.99
22 Kernel Bayesian	9.3	9.39	6.42	6.42	0.96	0.96
23 Ensemble Bayesian	6.38	5.75	3.51	2.92	0.98	0.99

Table 9S. Details of the predictive models; prediction speed, training time and model size.

Prediction Model	Prediction Speed (obs/msec)	Training Time (sec)	Model Size (kilobytes)
1 Linear Regression	15.34	13.73	7.26
2 Robust Linear Regression	36.12	47.98	7.8
3 Stepwise Linear Regression	41.07	46.33	8.82
4 Fine Tree	72.05	44.87	61.95
5 Medium Tree	67.59	43.44	30.82
6 Coarse Tree	68.99	42.2	11.97
7 Linear SVM	77.12	40.83	64.68
8 Qudratic SVM	77.03	39.71	44.4
9 Cubic SVM	29.31	37.79	34.12
10 Fine Gaussian SVM	40.21	36.19	46.36
11 Medium Gaussian SVM	17.99	34.92	30.42
12 Coarse Gaussian SVM	26.78	26.55	42.38
13 Boosted Trees Ensemble	39.31	3.45	161.29
14 Bagged Trees Ensemble	16.32	3.3	1125.27
15 Squared Exponential GPR	14.33	64.07	98.25
16 Maten 5/2 GPR	13.62	65.39	98.23
17 Exponential GPR	9.66	73.29	98.23
18 Rational Quadratic GPR	11.65	157.23	98.28
19 Tree Bayesian	52.01	114.49	69.94
20 SVM Bayesian	18.8	878.46	90.96
21 GPR Bayesian	21.13	3496.57	98.44
22 Kernel Bayesian	63.99	95.17	10.32
23 Ensemble Bayesian	23.66	164.89	1015.27

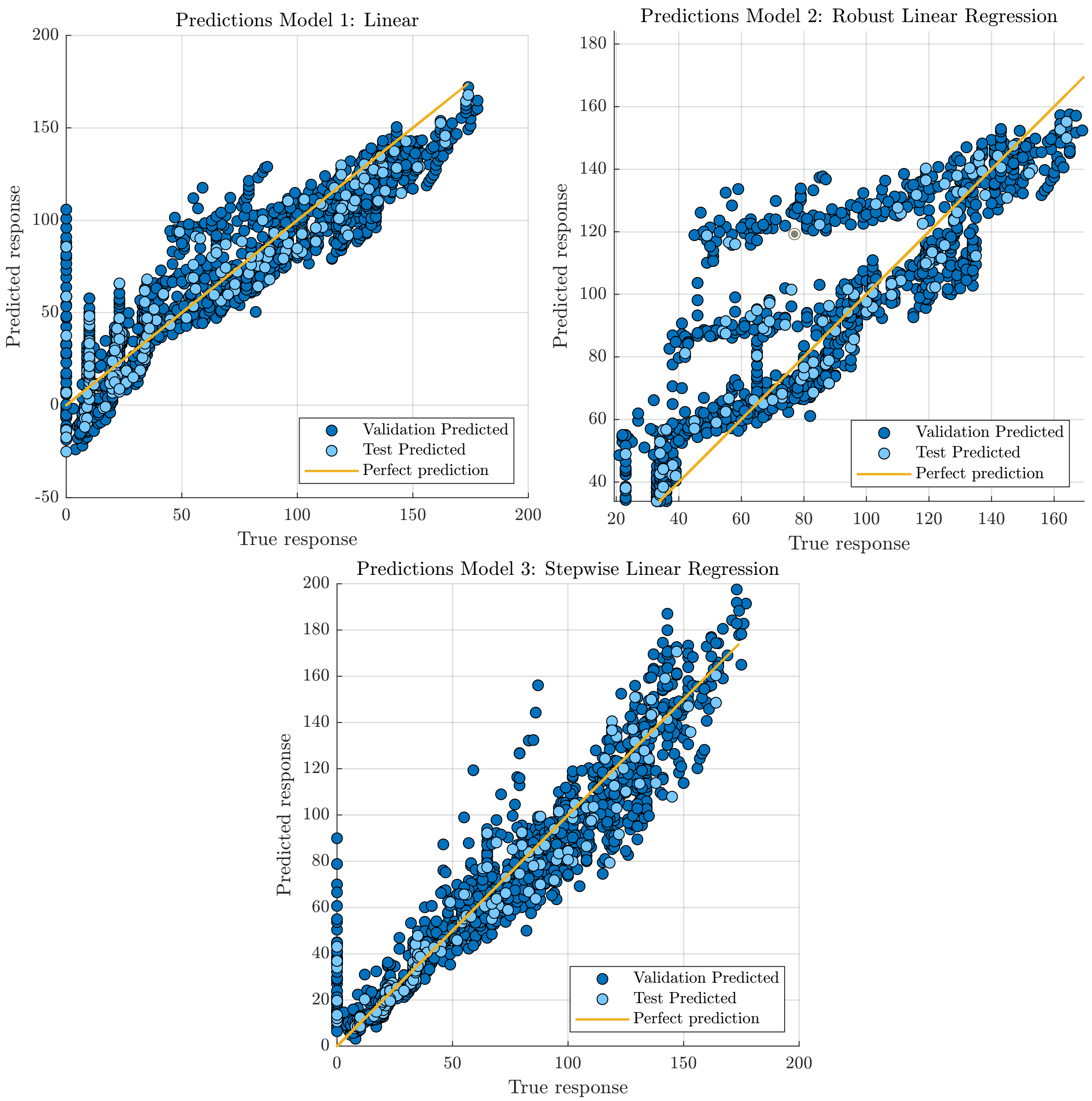

Figure 4S. The result of Linear prediction models; prediction response versus the true responses for Linear, Robust and Step-wise Linear Regression models.

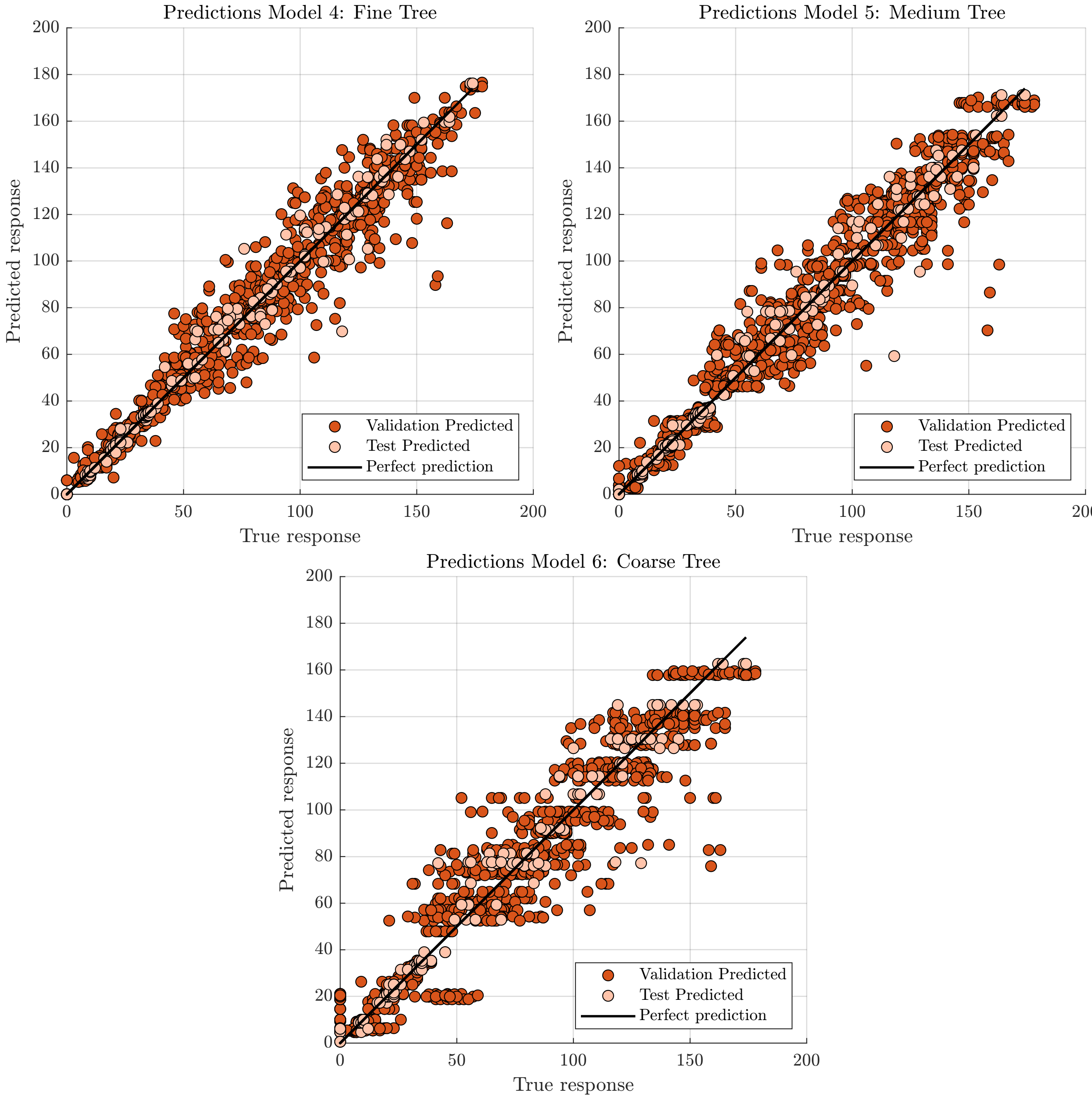

Figure 5S. The result of Tree predictions models; prediction response versus the true responses for Fine, Medium, and Coarse Tree models.

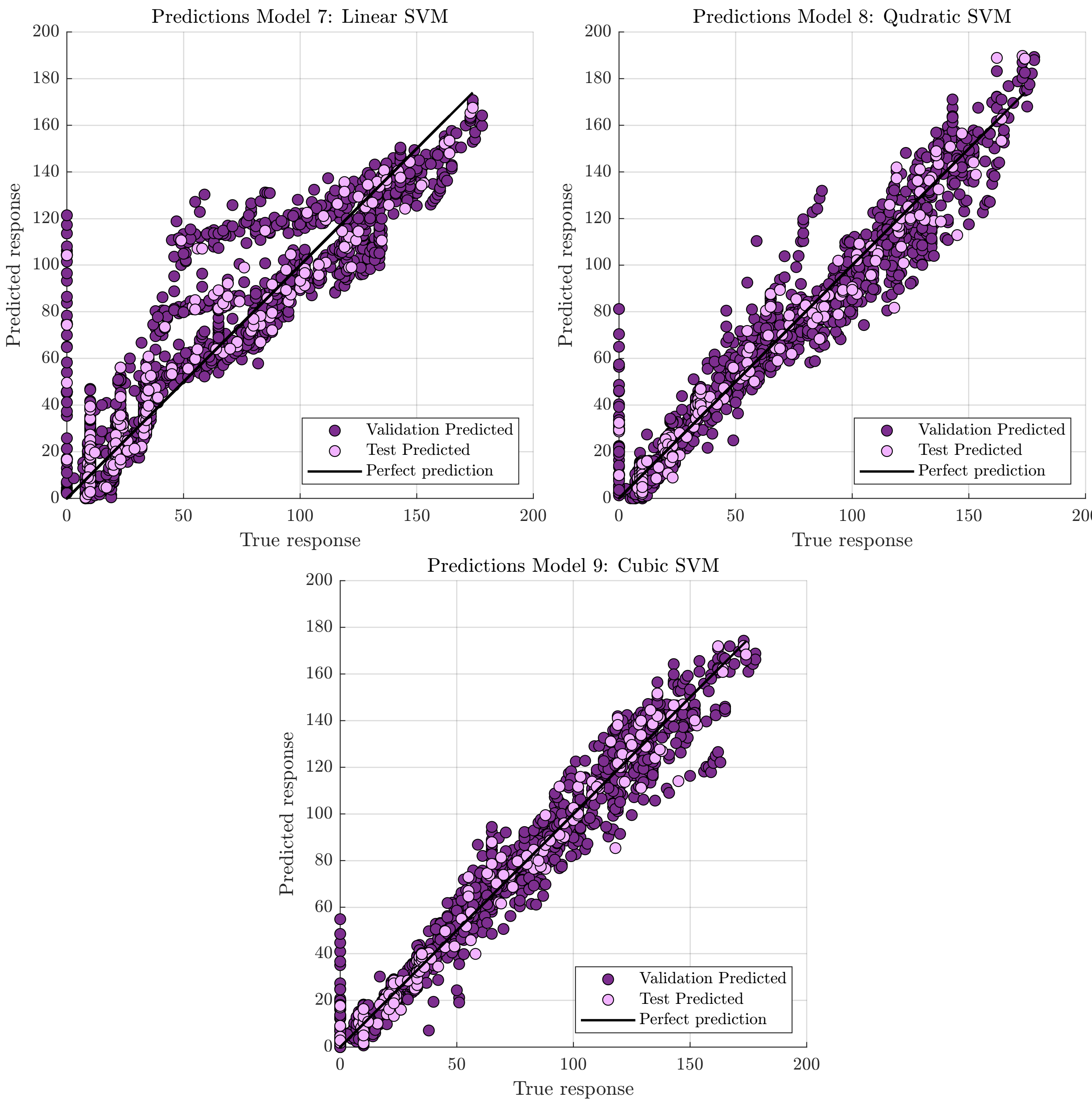

Figure 6S. The results of SVM prediction models; prediction response versus the true responses for Linear, Quadratic and Cubic SVM models.

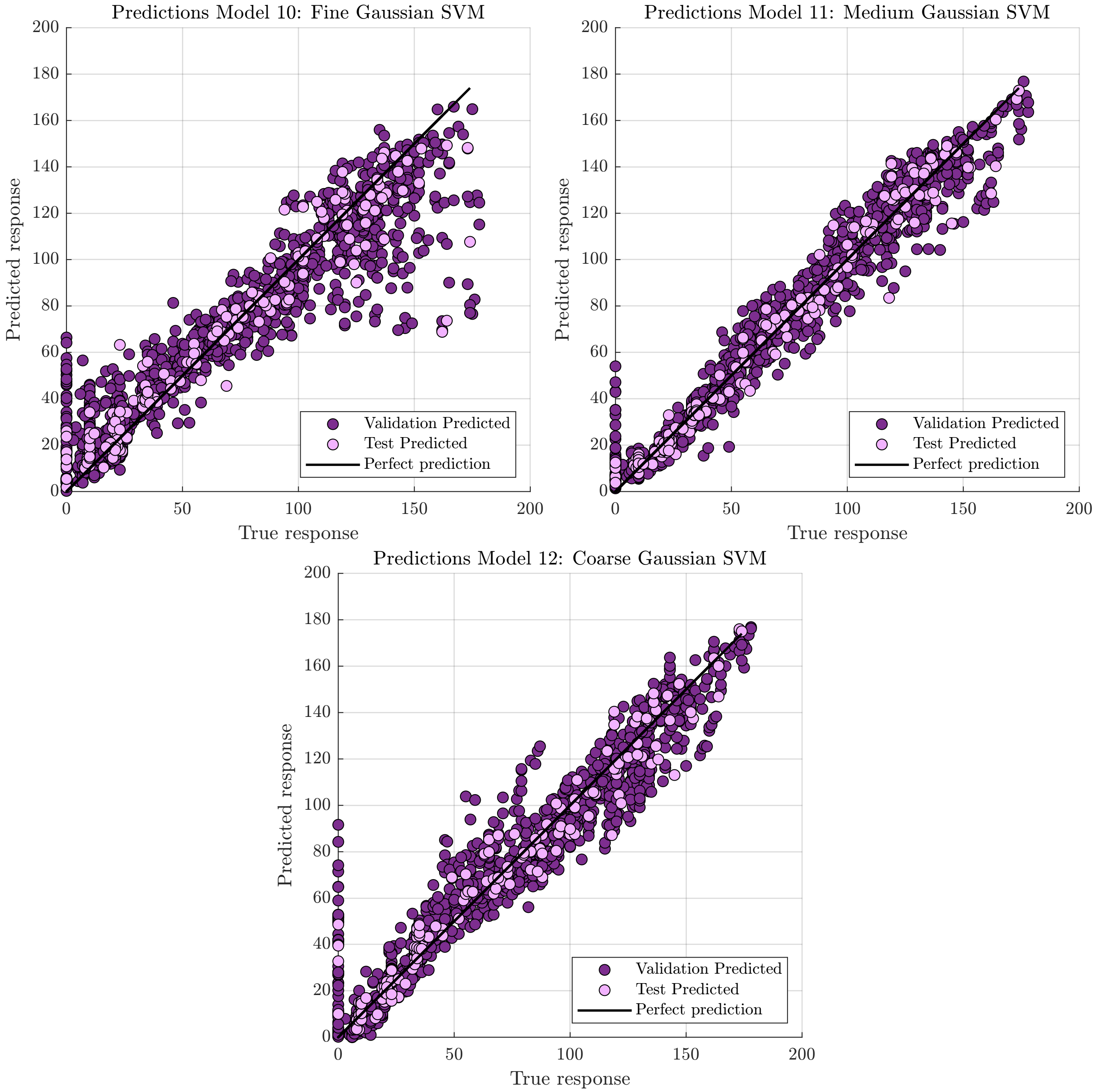

Figure 7S. The results of SVM Gaussian prediction models; prediction response versus the true responses for Fine, Medium and Coarse Gaussian SVM models.

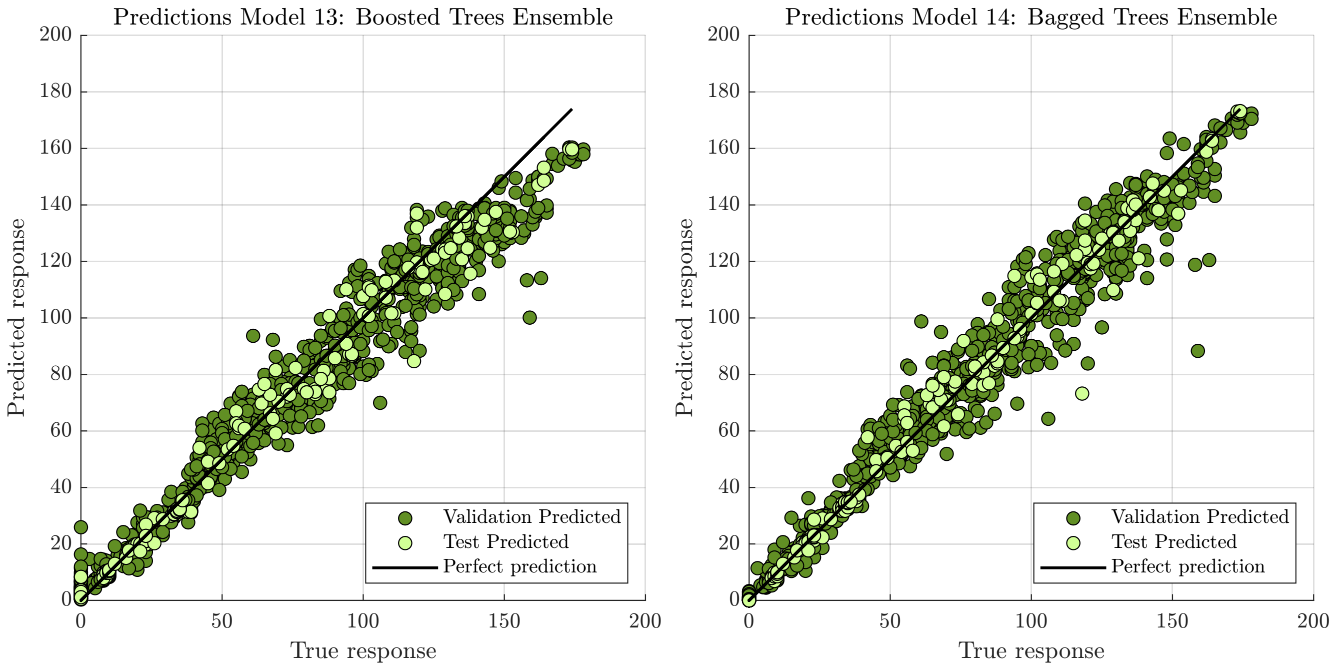

Figure 8S. The results of Ensemble predictions models; prediction response versus the true responses for Boosted and Bagged Trees Ensemble models.

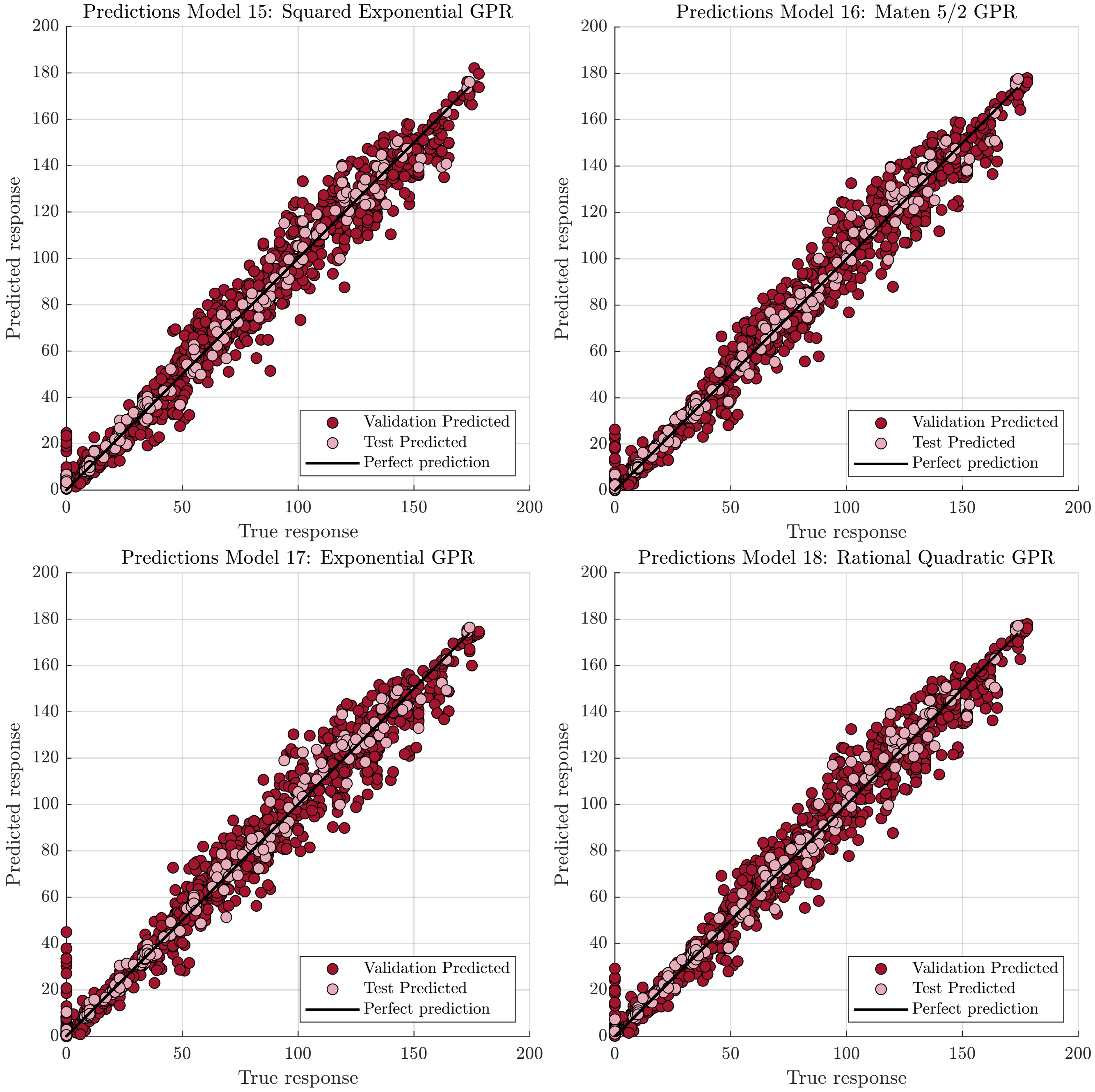

Figure 9S. The results of GPR and Ensemble predictions models; prediction response versus the true responses for Squared Exponential, Matern 5/2, Exponential and Rational Quadratic GPR models.

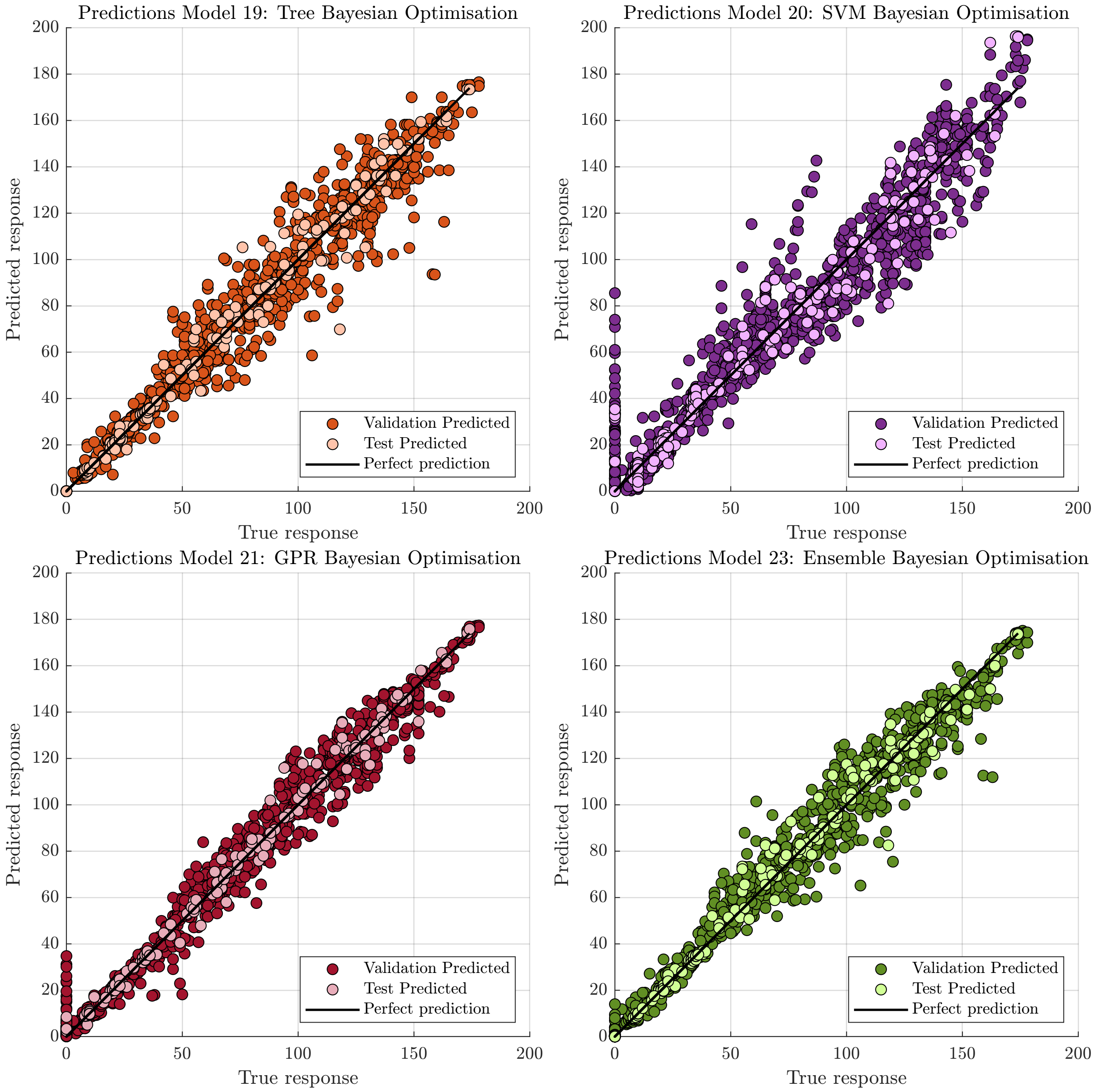

Figure 10S. The results of Bayesian optimisation based predictions models; for Trees, Ensemble, SVM and GPR.
